# Comparative Elucidation of Age, Diameter, and “Pockmarks” in Roots of *Paeonia lactiflora* Pall. *(Shaoyao)* by Qualitative and Quantitative Methods

**DOI:** 10.3389/fpls.2021.802196

**Published:** 2022-01-26

**Authors:** Xiaowen Zheng, Minzhen Yin, Shanshan Chu, Mei Yang, Zhengyang Yang, Yuejian Zhu, Luqi Huang, Huasheng Peng

**Affiliations:** ^1^School of Pharmacy, Anhui University of Chinese Medicine, Hefei, China; ^2^National Resource Center for Chinese Materia Medica, China Academy of Chinese Medical Sciences, Beijing, China; ^3^Anhui Province Key Laboratory of Research & Development of Chinese Medicine, Hefei, China; ^4^Jiren Pharmaceutical, Bozhou, China; ^5^Research Unit of DAO-DI Herbs, Chinese Academy of Medical Sciences, 2019RU57, Beijing, China

**Keywords:** *Paeonia lactiflora* Pall., age, diameter, pockmarks, quality evaluation

## Abstract

*Paeonia lactiflora* Pall. is a world-famous ornamental plant, whose roots have been used as an important traditional Chinese medicine, *Shaoyao*, to treat diseases for more than 1,000 years. Because of the excellent curative effect of *Shaoyao*, its quality has attracted wide attention, however, there is a lack of comprehensive research on the different influencing factors of quality of *Shaoyao*. In this study, ultra-performance liquid chromatography-quadrupole time-of-flight mass spectrometry (UPLC-Q/TOF-MS) and high-performance liquid chromatography with diode-array detection (HPLC-DAD) were utilized to systematically analyze the *Shaoyao* of different ages, diameters and roots with “pockmarks.” 60 metabolites were detected and identified from *Shaoyao* using the UPLC-Q/TOF-MS, of which 20 potential quality markers of dissected roots with and without “pockmarks” were selected for the first time using the orthogonal partial least squares discriminant analysis (OPLS-DA) and the variable importance for projection (VIP) plot. Then, a selective and accurate HPLC-DAD quantitative assay has been developed for the simultaneous determination of 11 bioactive components in *Shaoyao*. The results showed that the total content of five monoterpene glycosides including oxypaeoniflorin, albiflorin, paeoniflorin, lactiflorin, and benzoylpaeoniflorin and six phenols including gallic acid, catechin, methyl gallate, ethyl gallate, apiopaeonoside and benzoic acid in the 3-year-old *Shaoyao* was higher than that of 4-year-old and 5-year-old *Shaoyao*. In *Shaoyao* of the same age, the total content of five monoterpene glycosides and six phenols decreased with an increase in diameter. In addition, regardless of whether it is a whole or a divided root, the contents of five monoterpene glycosides and six phenols in *Shaoyao* with “pockmarks” were higher than those of *Shaoyao* without “pockmarks.” In summary, this work has explored several factors that might affect the quality of *Shaoyao*, and provide a guide for more comprehensive quality evaluation in its further production, processing, and rational utilization.

## Introduction

*Shaoyao*, derived from the root of *Paeonia lactiflora* Pall., is one of the most common traditional Chinese medicines. It was first recorded in *Shen Nong’s Herbal Classic*, and has been used for nearly 1000 years (Yang et al., 2020). Modern phytochemical studies have shown that monoterpene glycosides, flavonoids, tannins, stilbenes, triterpenoids, steroids, and phenols are the major biologically active components of *P. lactiflora* (Tan et al., 2020; Li P. et al., 2021). The compounds and extracts obtained from this herbal medicine have been reported to exhibit an extensive range of biological functions, including anti-inflammatory (Ma et al., 2018), cardioprotective (Liu et al., 2019), neuroprotective (Li et al., 2018), and hepatoprotective (Li et al., 2018; Xie et al., 2018) activities. Additionally, it can be also used as antidepressants (Cheng et al., 2021) and in migraine treatment (Liao et al., 2019).

*Paeonia lactiflora*, a perennial herb of the Paeoniaceae family, is widely distributed in China, Japan, South Korea, Mongolia, and Russia (Far East Siberia) (Wu et al., 2001). It’s widely cultivated as an ornamental plant for medicinal use. Currently, Bozhou City (Anhui Province), Pan’an County (Zhejiang Province), Zhongjiang County (Sichuan Province), and Heze City (Shandong Province) are the four main areas of *P. lactiflora* (Zha et al., 2011). Among them, *Shaoyao* from Bozhou City is known as one of the “authentic medicinal materials in Anhui” because of its excellent texture, high medicinal value, and large output (Peng et al., 2017a).

**FIGURE 1:**
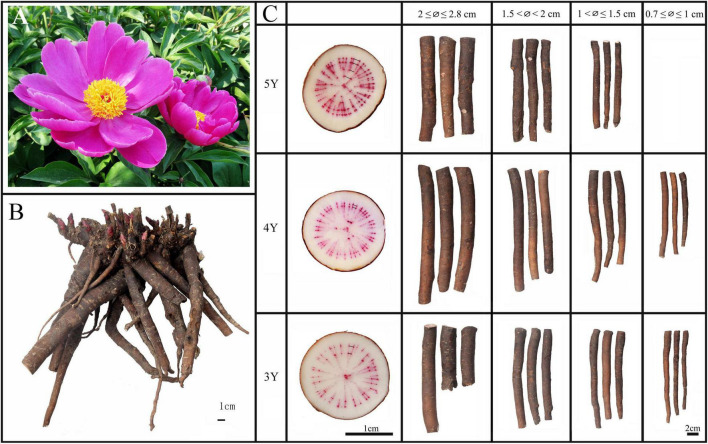
Characteristics of flower **(A)**, root system **(B)**, and roots of different ages and diameter of *P. lactiflora* **(C)**. 3Y, 3-year-old roots of *P. lactiflora*; 4Y, 4-year-old roots of *P. lactiflora*; 5Y, 5-year-old roots of *P. lactiflora*; ∅, median diameter of *P. lactiflora* roots.

**FIGURE 2:**
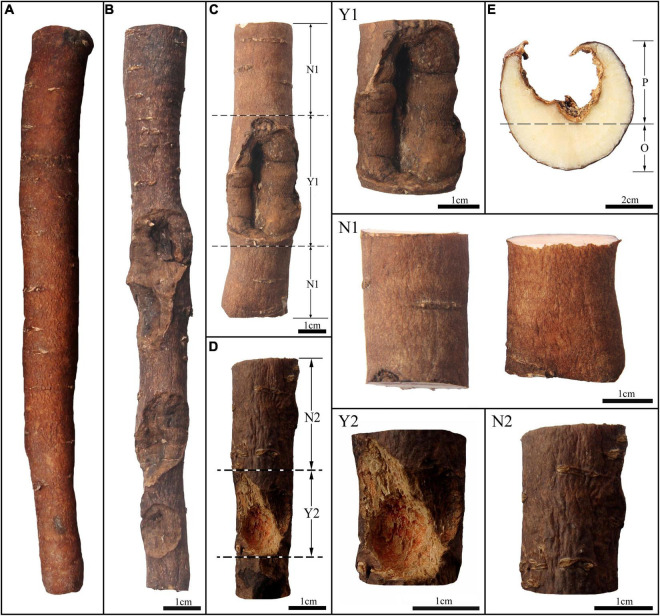
Morphological characters of *P. lactiflora* roots with and without “pockmarks.” *P. lactiflora* root without “pockmarks” **(A)** and with “pockmarks” **(B)**; root with old formed “pockmarks” **(C)**; root with newly formed “pockmarks” **(D)**; divided parts with (Y1) and without (N1) old formed “pockmarks” in the same root; divided parts with (Y2) and without (N2) newly formed “pockmarks” in the same root; transverse section of *P. lactiflora* root with “pockmarks” **(E)**, P, the part with “pockmarks” and O, the part without “pockmarks.”

In previous field investigations, we found that *Shaoyao* from Bozhou was generally harvested after 3–5 years of growth. The root system of *P. lactiflora* develops from adventitious roots, so roots of different age and diameter coexist in the same root system when harvested (Figure 1). Although early studies have found that the quality of *Shaoyao* is related to the age and diameter (Zha et al., 2012; Wang et al., 2015), the division of age and diameter is sketchy, how it affects remains controversial and requires further study. In addition, after years of cultivation, the roots of *P. lactiflora* often suffer damage due to chewing by insects such as *Holotrichia diomphalia* Bates and *Gryllotalpa unispina* Saussure, causing the formation of calluses in wounded site on surface of the roots (Figure 2; Leitner et al., 2005). Calluses formed in wounded site that look like scars are often called “pockmarks.” Studies have shown that the types and contents of chemical compounds will be affected after the attack of insects (Leitner et al., 2005; Wang et al., 2018), but there is no report on the impact of insects chewing on the chemical compounds in roots of *P. lactiflora*. Therefore, whether the “pockmarks” caused by insect chewing has an impact on quality of *Shaoyao* still needs further research.

Previous studies have mainly focused on the phytochemistry, pharmacological evaluation, pharmacokinetics, clinical application and quality evaluation of *Shaoyao* (Du et al., 2020; Zhang and Wei, 2020). However, the factors affecting the quality of *Shaoyao* investigated in previous researches are relatively single, so a more comprehensive research on the different influencing factors of its quality is necessary (Wang et al., 2015; Li and Yuan, 2017). In this study, the age of *Shaoyao* was accurately identified by the growth rings based on previous study (Chu et al., 2018), and the differences of main active ingredients in *Shaoyao* with different ages, diameters, and “pockmarks” were systematically clarified by ultra-performance liquid chromatography quadrupole time-of-flight mass spectrometry (UPLC-Q/TOF-MS) qualitative analysis and high-performance liquid chromatography with diode-array detection (HPLC-DAD) quantitative analysis. These results will provide a scientific foundation for further improving the quality evaluation and rational utilization of *Shaoyao*.

## Materials and Methods

### Plant Materials

Twenty-eight groups of *P. lactiflora* roots were collected from Bozhou City, Anhui Province, China, and all roots were authenticated by Prof. Huasheng Peng from the National Resource Center for Chinese Materia Medica, China Academy of Chinese Medical Sciences. All the roots were accurately identified by the growth rings for their growth years based on previous study (Figure 1C; Zha et al., 2011; Chu et al., 2018). Among them, 9 groups of samples were used for further study on different ages, with 15–20 individuals in each group. Other samples include 11 groups of samples used for study on different diameters and 8 groups of samples used for study on “pockmarks,” with detailed sample information provided in Supplementary Table 1. Among the 8 groups of samples used for study on “pockmarks,” 4 groups of samples were the whole roots almost covered with “pockmarks.” And for other samples, some old or newly formed “pockmarks” were occasionally distributed on the surfaces of roots, which were divided as shown in Figures 2C,D. The roots with some old “pockmarks” were divided into parts with and without “pockmarks” (Figure 2C), and roots with some newly formed “pockmarks” were also divided into two parts (Figure 2D). In order to evaluate the impact of “pockmarks” more comprehensively, the parts with “pockmarks” were cut longitudinally as shown in Figure 2E. All samples were dried and ground into powders (65 mesh), and then stored under dry conditions at room temperature before analysis.

### Chemicals and Reagents

The standard compounds paeoniflorin, albiflorin, catechin, lactiflorin, benzoylpaeoniflorin, 1,2,3,4,6-*O*-pentagalloylglucose, ethyl gallate, 3,4-dihydroxybenzaldehyde and 3,4-dihydroxybenzoic acid were purchased from Shanghai Yuanye Bio-Technology Co., Ltd. (Shanghai, China). Gallic acid, oxypaeoniflorin, methyl gallate, benzoic acid, and apiopaeonoside were obtained from Chengdu Push Bio-Technology Co., Ltd. (Chengdu, China). The purity of each standard was >98%. The chemical structures of the 14 reference compounds were presented in Supplementary Figure 1. A Milli-Q system (Millipore, MA, United States) was used to prepare ultra-pure water. HPLC-grade acetonitrile and methanol were procured from Tedia (Fairfield, OH, United States), and formic acid (LC-MS grade) was purchased from Aladdin (California, United States). All other reagents were of analytical grade.

### Standard and Sample Preparation

The standard compounds were accurately weighed and dissolved in methanol (v/v) to prepare stock solutions of approximately 1.0 mg/mL. Each stock solution was further diluted with methanol to prepare a series of concentrations, which were used to construct a calibration curve. The standard solutions were stored at –20°C and filtered with a Millipore filter (0.45 μm) before injection.

The powdered samples (0.2 g) were accurately weighed and dissolved in 10 mL of 60% methanol (v/v). The mixtures were extracted by ultrasonication (40 kHz, 200 W) at 30°C for 30 min after accurate weighting. The solutions were then cooled to room temperature, and the same solvent was added to each solution to compensate for the lost weight. The solutions were subsequently filtered through a Millipore filter (0.45 μm), and all the solutions were stored at –20°C for HPLC and UPLC-Q/TOF-MS analysis.

### Ultra-Performance Liquid Chromatography-Quadrupole Time-of-Flight Mass Spectrometry Conditions for Qualitative Analysis

The UPLC separation was performed at 30°C on a Waters Acquity UPLC system (Waters Corp., Milford, MA, United States), equipped with an Acquity UPLC BEH C18 column (100 mm × 2.1 mm, 1.7 μm) and a BEH C18 VanGuard pre-column (2.1 mm × 5 mm, 1.7 μm). Acetonitrile (A) and water containing 0.1% formic acid (B) formed the mobile phase system. The UPLC gradient elution condition was as follows: 0–1 min, 5% A; 1–2 min, 5–10% A; 2–15 min, 10–40% A; 15–20 min, 40–65% A; 20–26 min, 65–95% A; 26–30 min, 95–5% A. The injection volume was 2 μL, and the flow rate was set at 0.2 mL/min. Mass spectrometry detection was performed on a Waters Xevo G2 -XS QTof Mass spectrometer (Waters Corp., Milford, MA, United States), which was equipped with an electrospray ionization source (ESI). ESI-MS data were acquired in the negative ion scan mode (ESI^–^), and the mass scan range was set at *m/z* 50–1200 Da. Leucine-enkephalin was used to ensure the reference lock quality. The following source parameters were used: capillary voltage, 2.5 kV; sample cone voltage and collision energy, 40 and 15 V, respectively; desolvation temperature, 350°C; source temperature, 100°C; cone gas flow rate, 50 L/h; and desolvation gas flow rate, 800 L/h.

From a search of PubMed, CNKI, and other databases, the documented chemical substances in *P. lactiflora* and its homologous species were summarized in a Microsoft Office Excel table to establish a database, which included the name, molecular formula, and molecular weight of each chemical substance, as well as MS/MS fragment ions.

### High-Performance Liquid Chromatography Conditions for Quantitative Analysis

An Agilent 1260 Infinity HPLC system (Agilent Technologies Inc., Santa Clara, CA, United States) was used for HPLC analysis. The system was composed of a binary pump, a degasser, an autosampler, a column heater, and a diode array detector. An Agilent ZORBAX Eclipse XDB-C18 column (250 mm × 4.6 mm, 5 μm) was used for chromatographic separation. The column temperature was maintained at 30°C for all analyses. The mobile phase consisted of acetonitrile (A) and water with 0.1% formic acid (B). The gradient elution condition was optimized as follows: 0–6 min, 10–15% A; 6–25 min, 15–28% A; 25–28 min, 28–40% A; 28–40 min, 40–70% A; with 2 min of balance back to 10% A. The flow rate was set at 1 mL/min, the injection volume was 10 μL, and the UV detection wavelength was maintained at 230 nm.

### Validation of the Quantitative Method

To construct the calibration curves, the stock standard solutions were first diluted to six different concentrations, and the relationship between the peak area and the concentration of the analyte was plotted. The limits of detection (LODs) and limits of quantitation (LOQs) were determined using serially diluted standard solutions, with signal-to-noise ratios (*S/N*) of 3 and 10 for the LODs and LOQs, respectively. The intra- and inter-day precisions for detection of the 11 analytes were evaluated in six replicates within 1 day and three consecutive days, respectively. Six replicates of sample No. 1 were used to verify the reproducibility of the method. The solutions were then analyzed at 0, 2, 4, 8, 12, and 24 h to determine the stability. Depending on the analyte content of the sample, each reference was added to the nine samples at different concentrations (80, 100, and 120%). The average recovery rates of the 11 chemical compounds were then determined.

### Data Preprocessing and Statistical Analysis

The Progenesis QI software (Ver. 3.3.1, Waters Co., Milford, MA, United States) was used to pretreat and identify potential markers from the MS*^E^* raw data, which was collected using MassLynx software (Ver. 4.1, Waters Co., Milford, MA, United States). A variety of multiple adduct ions, including [M + HCOOH-H]^–^, [2M + Hac-H]^–^, [M + FA-H]^–^, [2M-H]^–^, [2M + FA-H]^–^, [M + H_2_O-H]^–^, [M-H]^–^, and [M-2H]^2–^ were selected or self-edited to remove redundant adduct ion species. Additionally, the apex peak detection and alignment processing algorithms were applied for further processing. The intensity of each ion was normalized according to the total ion count to generate a marker that consisted of the retention time, normalized peak area, and *m/z* value (Zhan et al., 2018). According to the Metabolomics Standards Initiative (Schymanski et al., 2014), reference standards with MS and retention time data can be used to derive the structural information at confidence level 1 (CL1), whereas other metabolite characteristics can be putatively identified at confidence level 2 (CL2). The normalized peak area and peak number ID (RT and *m/z* pair) were imported to the SIMCA 14.1 software (Ver. 2.0, Umetrics, Malmo, Sweden) for multivariate statistical analysis, including supervised orthogonal partial least squares discrimination analysis (OPLS-DA) and partial least squares discriminant analysis (PLS-DA). TBtools software (Ver. 1.09854, CHN) was used to generate a heatmap based on a hierarchical clustering analysis. In addition, GraphPad Prism software (Ver. 8.0.1, GraphPad, San Diego, CA, United States) was used to analyze the histogram for ANOVA. Correlation analysis was performed using Origin2021 software (Ver. 9.8.5, OriginLab, United States).

## Results

### Identification of Major Chemical Components in the Roots of *P. lactiflora* by Ultra-Performance Liquid Chromatography-Quadrupole Time-of-flight Mass Spectrometry

**FIGURE 3:**
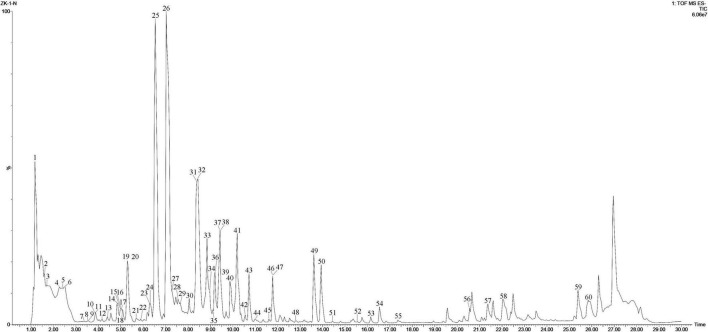
The TIC chromatogram of *P. lactiflora* root in negative mode.

The *P. lactiflora* roots of different ages, diameters and “pockmarks” were analyzed by UPLC-Q/TOF-MS under the optimized conditions. The total ion chromatogram (TIC) of *P. lactiflora* root was shown in Figure 3. Based on the high-resolution MS/MS data, ratios, retention time, elution order, mass spectra, and comparison with the reference standards, a total of 60 compounds were tentatively identified in the negative mode: 25 monoterpene compounds and their glycosides (peaks 6, 8, 10, 12, 14, 19, 25–26, 32–34, 36, 38, 43, 45, 47–55, and 57), 16 tannins (peaks 3, 5, 7, 9, 13, 15–16, 22–23, 27–28, 31, 35, 37, 40, and 46), 14 phenols (peaks 2, 4, 11, 17–18, 20–21, 24, 29–30, 39, 41–42, and 44), and 5 other compounds (peaks 1, 56, and 58–60). The detected and identified compounds were summarized in Supplementary Table 2, which were numbered according to the elution order. The MS data of 14 reference compounds (peaks 4, 11, 14, 17–18, 20, 24–26, 29, 31, 39, 43, and 49) were shown in Supplementary Figure 2.

#### Monoterpene Compounds and Their Glycosides

Twenty-five peaks were identified as monoterpene and their glycosides. Electrospray ionization mass spectrometry (ESI-MS) showed that the fragmentation patterns of these compounds were similar. Paeoniflorin (peak 26) and its isomers albiflorin (peak 25) were chosen as examples to analyze fragmentation patterns of monoterpene and their glycosides. After comparing albiflorin and paeoniflorin to the corresponding reference substance, it was found that the peak of albiflorin appeared earlier than that of paeoniflorin. In the negative ion mode, [M-H + HCOOH]^–^ at *m/z* 525.1614 and [M-H]^–^ at *m/z* 479.1546 both support the molecular formula C_23_H_28_O_11_. The fragment ion peaks at *m/z* 449.1503 and 327.1139 were generated from the neutral loss of formaldehyde (CH_2_O, 30 Da) and benzoic acid (C_7_H_6_O_2_, 122 Da). The fragment ion at *m/z* 357.1258 was formed by the loss of benzoic acid from the molecular ion [M-H]^–^. The fragment at *m/z* 165.0588 [M-H-C_8_H_7_O_2_-C_6_H_11_O_6_]^–^ was associated with the “cage-like” pinene skeleton.

#### Tannins

Sixteen peaks were identified as tannins. The MS fragmentation patterns of these compounds shared some common characteristics. For example, several galloyl moieties were lost (C_7_H_4_O_4_, 152 Da), and the gallic acid (C_7_H_6_O_5_, 170 Da) fragment ion at *m/z* 169 was a characteristic fragment ion. However, owing to the limited information, the connecting position of galloyl groups cannot be determined. Peaks 35 and 37, associated with hexagalloyl glucose, produced [M-H]^–^ ion at *m/z* 1091.1292, [M-H-galloyl]^–^ ion at *m/z* 939.1392, and [M-H-galloyl-gallic acid]^–^ ion at *m/z* 769.1083. Peak 27, associated with tetragalloyl glucose, produced [M-H]^–^ ion at *m/z* 787.1058. The fragment ions [M-H-galloyl]^–^, [M-H-gallic acid]^–^, [M-H-galloyl-gallic acid]^–^, and [M-H-2galloyl-gallic acid]^–^ produced peaks at *m/z* 635.1034, *m/z* 617.0904, *m/z* 465.0752, and *m/z* 313.0597, respectively. Similarly, the molecular ion at *m/z* 635.0879 [M-H]^–^ and the fragment ions at *m/z* 465.0708 and *m/z* 313.0597 were used to identify peaks 15 and 22 as trigalloyl glucose.

#### Phenols

Peaks 4, 11, 17, 18, 20, 24, 29, and 39 were identified as gallic acid, 3,4-dihydroxybenzoic acid, 3,4-dihydroxybenzaldehyde, catechin, methyl gallate, apiopaeonoside, ethyl gallate, and benzoic acid, respectively, by comparison with the corresponding standards. Peak 39 displayed a molecular ion [M-H]^–^ at m/z 121.0287 and a fragment ion at m/z 103.6157 [M-H-H_2_O]^–^, to identify benzoic acid. The molecular ion [M-H]^–^ of peak 20 was detected at *m/z* 183.0327, and then yielded diagnostic ion at *m/z* 168.0085 by concurrently losing a neutral CH_3_. Combined with the fragment ion at *m/z* 124.0185 [M-H-C_2_H_3_O_2_]^–^, it was speculated that peak 20 was methyl gallate. Similarly, peak 29 was identified as ethyl gallate, with an [M-H]^–^ ion at *m/z* 197.045, and an [M-H-C_2_H_4_] ^–^ ion at *m/z* 168.0085.

### Quantitative Analysis of the Main Chemical Components in *P. lactiflora* Roots of Different Ages and Diameters

#### Method Validation for Quantitative Analysis

A total of 11 chemical compounds in the roots of *P. lactiflora*, including paeoniflorin, oxypaeoniflorin, albiflorin, benzoylpaeoniflorin, gallic acid, methyl gallate, catechin, apiopaeonoside, lactiflorin, ethyl gallate, and benzoic acid were quantitatively and simultaneously determined. As summarized in Table 1, the linearity, LOD, LOQ, precision, repeatability, stability, and recovery were determined for method validation. The analyte concentrations and peak areas demonstrated good linearity (*R*^2^ > 0.999) within the test ranges. The intra- and inter-day precisions for the 11 analytes (RSDs) were within 0.84–1.90 and 0.95–2.65%, respectively. The average recovery of the analytes was in the range of 96.38–103.65% with RSDs lower than 3.32%. The results showed that the quantitative method was accurate and reliable for simultaneously determining the 11 target compounds.

**TABLE 1:** Method validation of 11 reference compounds.

Analytes	Calibration curves	*r* ^2^	Linear range (ug/mL)	LOD (ug/mL)	LOQ (ug/mL)	Precision (RSD, %)		Repeatability (RSD, %)	Stability (RSD, %)	Recovery	
						Intra-day	Inter-day			Mean, %	RSD, %
Paeoniflorin	y = 16320x−260.69	0.9993	5–1130	0.328	1	0.87%	1.80%	0.85%	0.71%	101.43	2.63
Albiflorin	y = 11455x + 78.695	0.9997	10–1197	1.09	4.25	1.03%	1.48%	0.60%	0.65%	100.55	2.7
Benzoic acid	y = 59122x + 0.45	0.9997	0.97656–62.5	0.144	0.55	1.27%	0.95%	0.94%	0.31%	101.30	2.97
Gallic acid	y = 10373x + 0.4289	1	1.953–250	0.488	1.7	0.92%	2.18%	0.70%	0.54%	99.61	2.58
Methyl gallate	y = 14576x−97.129	0.9991	6.15–250	2.25	6.15	0.84%	1.83%	0.23%	1.74%	98.61	3.02
Ethyl gallate	y = 14993x + 13.33	0.9998	0.977–62.5	0.32	0.9765	0.97%	1.32%	1.71%	2.98%	98.51	3.21
Oxypaeoniflorin	y = 2269x−3.6691	0.9999	5–180	1.25	4.25	1.55%	1.77%	1.56%	1.30%	99.75	3.32
Lactiflorin	y = 13881x + 3.2585	0.9998	0.585–30	0.156	0.585	1.35%	2.02%	0.84%	1.02%	101.43	2.63
Apiopaeonoside	y = 11287x−3.7639	0.9999	1.7–125	0.55	1.7	1.49%	2.65%	1.17%	2.24%	100.90	2.41
Benzoylpaeoniflorin	y = 20608x−2.9863	0.9998	0.46875–40	0.117	0.585	1.90%	2.56%	0.53%	1.08%	98.46	2.48
Catechin	y = 13617x−24.706	0.9996	1.5–80	0.315	1.25	1.43%	2.62%	0.84%	1.65%	100.56	1.86

#### Comparative Analysis of Main Chemical Components in *Shaoyao* of Different Ages

**FIGURE 4:**
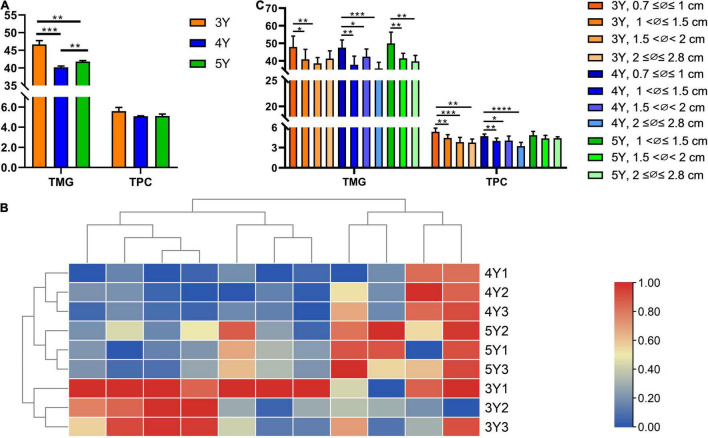
Contents of 11 reference compounds in *P. lactiflora* roots of different ages **(A,B)** and different diameters **(C)**. TMG, total content of five monoterpene glycosides; TPC, total content of six phenols. *p < 0.05, **p < 0.01, ***p < 0.001, ****p < 0.0001.

Further quantitative analysis was carried out to explore whether there were significant differences in the metabolite levels of *Shaoyao* of different ages. Simultaneous determination of 11 compounds in *Shaoyao* of different ages was conducted by HPLC (Supplementary Figure 3). The results were presented in Table 2 and Figure 4. The contents of 11 compounds showed differences in these samples. Paeoniflorin and albiflorin were of high content in all the samples. The total contents of five monoterpene glycosides including oxypaeoniflorin, albiflorin, paeoniflorin, lactiflorin and benzoylpaeoniflorin in roots of the 3-year-old *P. lactiflora* were 45.934–47.807 mg/g, whereas the total contents of six phenols including gallic acid, catechin, methyl gallate, ethyl gallate, apiopaeonoside and benzoic acid were 5.345–6.072 mg/g (Table 2). When compared with samples of other ages, the total content of these compounds in the 3-year-old *Shaoyao* was found to be relatively higher (Figure 4A). The content of paeoniflorin, an important quality control parameter in the market (Committee for the Pharmacopeia of P.R. China, 2020), was 28.378–28.689 mg/g in the 3-year-old *Shaoyao*, which was higher than that in *Shaoyao* of 4- or 5-year-old. The hierarchical clustering analysis heat map was used to compare the differences among the contents of the 11 compounds in *Shaoyao* of different ages (Figure 4B). The results showed that the 3-, 4-, and 5-year-old samples of *Shaoyao* were clearly separated at an appropriate distance level, and three biological replicates of each age were compactly gathered together, indicating that the experiment was reproducible and reliable.

**TABLE 2:** The content (mg/g) of 11 reference compounds in different ages, different diameters and “pockmarks” samples of *P. lactiflora*.

													
No.	Gallic acid	Oxypaeoniflorin	Catechin	Methyl gallate	Apiopaeonoside	Albiflorin	Paeoniflorin	Ethylgallate	Benzoic Acid	lactiflorin	benzoylpaeoniflorin	TMG	TPC
													
3Y1	1.916 ± 0.041	4.505 ± 0.761	0.92 ± 0.077	1.909 ± 0.069	0.527 ± 0.032	13.946 ± 0.003	28.378 ± 0.362	0.087 ± 0.02	0.71 ± 0.013	0.404 ± 0.124	0.571 ± 0.004	47.807 ± 0.999	6.072 ± 0.047
3Y2	1.778 ± 0.027	2.516 ± 0.173	0.687 ± 0.021	1.81 ± 0.023	0.251 ± 0.332	13.924 ± 0.143	28.689 ± 0.027	0.079 ± 0.101	0.738 ± 0.022	0.297 ± 0.019	0.51 ± 0.078	45.937 ± 0.402	5.345 ± 0.383
3Y3	1.801 ± 0.01	2.673 ± 0.123	0.639 ± 0.068	1.861 ± 0.014	0.502 ± 0.041	13.875 ± 0.039	28.615 ± 0.054	0.12 ± 0.12	0.719 ± 0.027	0.317 ± 0.016	0.451 ± 0.003	45.934 ± 0.088	5.645 ± 0.069
4Y1	1.91 ± 0.022	2.426 ± 0.155	0.616 ± 0.027	1.33 ± 0.005	0.479 ± 0.017	10.132 ± 0.259	26.612 ± 0.465	0.036 ± 0.016	0.727 ± 0.006	0.283 ± 0.037	0.295 ± 0.011	39.749 ± 0.831	5.1 ± 0.061
4Y2	1.944 ± 0.002	2.72 ± 0.035	0.601 ± 0.007	1.359 ± 0.006	0.488 ± 0.005	10.316 ± 0.025	26.517 ± 0.633	0.098 ± 0.077	0.728 ± 0.002	0.255 ± 0.001	0.351 ± 0.069	40.16 ± 0.643	5.22 ± 0.092
4Y3	1.911 ± 0.044	2.715 ± 0.096	0.595 ± 0.014	1.351 ± 0.028	0.505 ± 0.008	10.42 ± 0.109	26.617 ± 0.496	0.116 ± 0.074	0.726 ± 0.001	0.28 ± 0.027	0.311 ± 0.016	40.346 ± 0.658	5.207 ± 0.155
5Y1	1.739 ± 0.014	3.108 ± 0.087	0.671 ± 0.008	1.225 ± 0.003	0.503 ± 0.002	10.688 ± 0.1	26.972 ± 0.007	0.142 ± 0.001	0.795 ± 0.003	0.356 ± 0.006	0.354 ± 0.005	41.481 ± 0.022	5.078 ± 0.029
5Y2	1.849 ± 0.073	2.941 ± 0.271	0.613 ± 0.055	1.528 ± 0.045	0.52 ± 0.033	10.747 ± 0.269	27.588 ± 0.612	0.132 ± 0.01	0.805 ± 0.004	0.386 ± 0.044	0.349 ± 0.001	42.014 ± 0.655	5.45 ± 0.0004
5Y3	1.865 ± 0.002	3.136 ± 0.041	0.662 ± 0.008	1.274 ± 0.012	0.508 ± 0.003	10.457 ± 0.033	27.104 ± 0.424	0.154 ± 0.03	0.763 ± 0.0004	0.348 ± 0.003	0.352 ± 0.007	41.399 ± 0.437	5.229 ± 0.008
R1	2.198 ± 0.517	2.525 ± 1.258	0.425 ± 0.15	1.078 ± 0.271	0.449 ± 0.123	14.892 ± 3.926	29.525 ± 4.804	0.048 ± 0.012	1.148 ± 0.104	0.494 ± 0.174	0.525 ± 0.131	48.012 ± 6.216	5.3 ± 0.559
R2	2.168 ± 0.383	1.484 ± 0.38	0.285 ± 0.071	0.662 ± 0.15	0.254 ± 0.121	12.744 ± 4.99	25.909 ± 1.336	0.043 ± 0.021	1.021 ± 0.094	0.409 ± 0.117	0.36 ± 0.05	40.951 ± 5.702	4.393 ± 0.501
R3	2.075 ± 0.638	1.267 ± 0.467	0.2 ± 0.022	0.467 ± 0.054	0.227 ± 0.076	9.876 ± 2.665	26.702 ± 1.166	0.032 ± 0.021	0.81 ± 0.059	0.423 ± 0.095	0.36 ± 0.079	38.662 ± 3.277	3.78 ± 0.703
R4	2.124 ± 0.297	1.191 ± 0.288	0.177 ± 0.019	0.49 ± 0.084	0.215 ± 0.067	8.85 ± 1.711	30.359 ± 2.172	0.04 ± 0.023	0.706 ± 0.033	0.44 ± 0.231	0.48 ± 0.068	41.362 ± 4.444	3.714 ± 0.502
R5	2.153 ± 0.201	2.423 ± 0.513	0.342 ± 0.09	0.794 ± 0.172	0.304 ± 0.114	15.893 ± 3.78	28.346 ± 3.12	0.041 ± 0.011	1.067 ± 0.202	0.494 ± 0.138	0.398 ± 0.063	47.598 ± 4.383	4.662 ± 0.306
R6	2.146 ± 0.273	2.029 ± 0.508	0.254 ± 0.054	0.526 ± 0.101	0.232 ± 0.102	8.101 ± 1.11	26.867 ± 3.941	0.043 ± 0.019	0.801 ± 0.063	0.524 ± 0.097	0.253 ± 0.043	37.819 ± 4.946	3.961 ± 0.389
R7	2.102 ± 0.575	1.869 ± 0.626	0.283 ± 0.087	0.611 ± 0.126	0.199 ± 0.132	10.615 ± 3.011	29.024 ± 2.287	0.046 ± 0.02	0.8 ± 0.057	0.497 ± 0.225	0.381 ± 0.102	42.434 ± 4.426	3.997 ± 0.659
R8	1.658 ± 0.532	1.28 ± 0.308	0.193 ± 0.055	0.436 ± 0.035	0.2 ± 0.086	7.121 ± 2.372	26.576 ± 2.84	0.032 ± 0.013	0.696 ± 0.067	0.395 ± 0.091	0.345 ± 0.109	35.752 ± 3.473	3.186 ± 0.545
R9	2.615 ± 0.313	1.24 ± 0.545	0.251 ± 0.132	0.493 ± 0.062	0.23 ± 0.134	15.314 ± 4.642	32.374 ± 5.223	0.072 ± 0.035	1.186 ± 0.225	0.598 ± 0.149	0.457 ± 0.065	50.058 ± 6.43	4.777 ± 0.538
R10	2.525 ± 0.457	1.006 ± 0.205	0.195 ± 0.066	0.423 ± 0.036	0.218 ± 0.085	9.195 ± 1.166	30.217 ± 2.638	0.052 ± 0.028	0.945 ± 0.16	0.612 ± 0.119	0.416 ± 0.043	41.502 ± 2.91	4.308 ± 0.456
R11	2.537 ± 0.235	0.942 ± 0.216	0.156 ± 0.016	0.399 ± 0.039	0.192 ± 0.092	6.873 ± 1.629	30.672 ± 2.208	0.057 ± 0.043	1.058 ± 0.07	0.799 ± 0.116	0.444 ± 0.071	39.79 ± 3.413	4.344 ± 0.227
PW1	3.172 ± 0.775	2.939 ± 1.235	0.759 ± 0.32	1.165 ± 0.266	0.548 ± 0.064	19.017 ± 7.146	30.408 ± 2.186	0.15 ± 0.079	1.118 ± 0.08	0.62 ± 0.072	0.524 ± 0.119	53.512 ± 6.235	6.914 ± 1.251
PW2	3.128 ± 0.295	2.453 ± 0.591	0.44 ± 0.032	1.134 ± 0.339	0.59 ± 0.119	11.46 ± 2.064	34.052 ± 4.545	0.121 ± 0.056	1.087 ± 0.13	0.596 ± 0.131	0.662 ± 0.198	49.225 ± 5.081	6.503 ± 0.499
PW3	2.903 ± 0.169	2.827 ± 1.421	0.787 ± 0.359	1.034 ± 0.244	0.533 ± 0.054	14.455 ± 3.383	27.387 ± 3.728	0.091 ± 0.023	1.061 ± 0.039	0.61 ± 0.115	0.433 ± 0.049	45.713 ± 2.631	6.412 ± 0.28
PW4	3.002 ± 0.362	1.587 ± 0.463	0.377 ± 0.072	0.767 ± 0.086	0.413 ± 0.098	8.732 ± 1.984	26.953 ± 1.766	0.09 ± 0.013	0.925 ± 0.083	0.606 ± 0.037	0.365 ± 0.094	38.246 ± 3.857	5.578 ± 0.237
PY1	2.297 ± 0.74	2.614 ± 0.199	1.097 ± 0.163	3.945 ± 1.645	1.014 ± 0.737	13.124 ± 6.779	38.404 ± 2.1	0.094 ± 0.045	0.181 ± 0.104	0.248 ± 0.266	0.699 ± 0.101	55.092 ± 8.254	8.63 ± 0.908
PN1	2.816 ± 0.35	2.428 ± 0.607	0.967 ± 0.325	1.615 ± 0.43	0.502 ± 0.082	12.607 ± 6.436	34.975 ± 3.037	0.104 ± 0.028	0.332 ± 0.019	0.458 ± 0.236	0.488 ± 0.135	50.959 ± 10.085	6.34 ± 1.097
PY2	1.525 ± 0.654	3.286 ± 1.288	0.747 ± 0.214	4.803 ± 1.391	0.818 ± 0.678	12.011 ± 2.493	34.848 ± 4.091	0.074 ± 0.016	0.186 ± 0.077	0.016 ± 0.007	0.641 ± 0.246	50.804 ± 6.101	8.155 ± 2.589
PN2	2.188 ± 0.705	2.729 ± 0.744	0.725 ± 0.146	2.243 ± 1.199	0.476 ± 0.054	12.017 ± 0.649	32.339 ± 3.556	0.076 ± 0.016	0.28 ± 0.05	0.1 ± 0.067	0.447 ± 0.057	47.633 ± 3.974	5.992 ± 0.348
PP1	1.573 ± 0.29	3.469 ± 2.009	1.203 ± 0.554	7.145 ± 1.344	0.317 ± 0.132	12.36 ± 2.392	37.037 ± 2.081	0.148 ± 0.068	0.374 ± 0.107	0.079 ± 0.008	0.746 ± 0.057	53.693 ± 6.288	10.762 ± 1.755
PO1	1.276 ± 0.128	2.077 ± 0.894	1.139 ± 0.704	3.176 ± 0.378	0.365 ± 0.043	7.541 ± 1.461	35.432 ± 3.802	0.133 ± 0.091	0.328 ± 0.031	0.068 ± 0.045	0.523 ± 0.095	45.644 ± 3.356	6.419 ± 0.819
PP2	1.959 ± 0.816	3.419 ± 0.901	1.101 ± 0.72	5.213 ± 1.28	0.447 ± 0.32	13.141 ± 6.223	33.987 ± 6.293	0.097 ± 0.019	0.402 ± 0.053	0.101 ± 0.032	1.087 ± 0.315	51.737 ± 13.694	9.221 ± 0.817
PO2	1.326 ± 0.374	1.523 ± 0.492	0.728 ± 0.445	3.121 ± 0.811	0.367 ± 0.114	7.709 ± 2.477	31.102 ± 3.152	0.063 ± 0.024	0.299 ± 0.011	0.061 ± 0.008	0.637 ± 0.187	41.034 ± 6.275	5.906 ± 0.396

*TMG, total content of five monoterpene glycosides; TPC, total content of six phenols; 3Y, 3-year-old roots of P. lactiflora; 4Y, 4-year-old roots of P. lactiflora; 5Y, 5-year-old roots of P. lactiflora; R, samples of different diameters; PW, whole roots with “pockmarks”; PY1, divided roots with old formed “pockmarks”; PN1, divided roots without old formed “pockmarks”; PY2, divided roots with newly formed “pockmarks”; PN2, divided roots without newly formed “pockmarks”; PP, parts with “pockmarks” of the longitudinally cut root; PO, parts without “pockmarks” of the longitudinally cut root.*

#### Comparative Analysis of Main Chemical Components in *Shaoyao* of Different Diameters

The growth ring was used to identify 3–5-year-old *Shaoyao* samples (Figure 1C). Based on the median diameter (∅) of root, the collected samples were divided into 4 groups: 0.7 ≤ ∅ ≤ 1, 1 < ∅ ≤ 1.5, 1.5 < ∅ < 2, and 2 ≤ ∅ ≤ 2.8 cm (Figure 1C). The quantitative results showed that the total content of five monoterpene glycosides and six phenols were significantly higher in the 3- and 4-year-old *P. lactiflora* with root diameters of 0.7 ≤ ∅ ≤ 1 cm (Figure 4C). With the increase of age, the root diameters also increased, and there were no samples of 5-year-old *P. lactiflora* with a size of ∅ ≤ 1 cm. Additionally, the total content of five monoterpene glycosides and six phenols were higher in the roots of 5-year-old *P. lactiflora* with a diameter in the range of 1 < ∅ ≤ 1.5 cm.

### Comparative Analysis of Main Chemical Components in *P. lactiflora* Roots With and Without “Pockmarks”

#### Qualitative Analysis of *Shaoyao* With and Without “Pockmarks”

**FIGURE 5:**
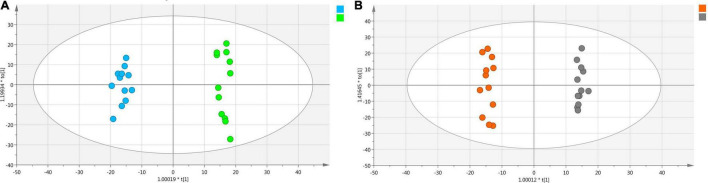
OPLS-DA analysis of whole roots with and without “pockmarks” **(A)** and the divided roots with and without “pockmarks” of *P. lactiflora* **(B)**. The blue dot represents the whole root with “pockmarks,” green dot represents the whole root without “pockmarks,” orange dot represents divided root without “pockmarks,” and gray dot represents divided root with “pockmarks.”

Optimized UPLC-Q/TOF/MS conditions were used to analyze *Shaoyao* samples with and without “pockmarks.” The TICs showed that the chemical constituents were similar between the two groups of *Shaoyao* samples, but there were differences in the metabolite level (Supplementary Figure 4). Therefore, the statistical analyses, such as PCA and OPLS-DA, were used to clarify the differences between *Shaoyao* samples with and without “pockmarks.” To ensure the repeatability and reliability of the data, we evaluated the data quality using a QC sample (Mais et al., 2018). The PCA results of all the samples were shown in Supplementary Figure 5A. The overlapping display and analysis of the mass spectrometry results of different samples and QC samples were separated and aggregated into three groups. Based on 200 random permutations, the R^2^-intercept was <0.4 and the Q^2^-intercept was <0, which indicated that the OPLS-DA model was valid without overfitting (Supplementary Figure 5B). The OPLS-DA result of *Shaoyao* with and without “pockmarks” has given an excellent model with *R*^2^ = 0.989 and *Q*^2^ = 0.980, and the plot showed complete separation of the two groups (Figure 5A). The total variation of the two groups was 57.2%, of which 36% was between samples with and without “pockmarks,” whereas 21.2% was within the group.

Additionally, potential quality markers between the divided roots with and without “pockmarks” (Figure 2C) were further screened. The OPLS-DA result of the two groups has given an outstanding model with *R*^2^ = 0.954 and *Q*^2^ = 0.88, and the plot showed definite groupings (Figure 5B). The total variation of the two groups was 48.6%. The differentiating contributions of each classification were ranked based on their VIP values. Variables with VIP values > 6 were considered as markers with high discrimination potential, and 20 potential markers were tentatively identified (Supplementary Figures 5E,F and Supplementary Table 3).

#### Quantitative Analysis of *Shaoyao* With and Without “Pockmarks”

To obtain accurate data on the differences between the roots of *P. lactiflora* with and without “pockmarks,” the main monoterpene glycosides and phenolic components in the whole and divided roots were determined and compared. First of all, the 4 groups whole roots of *P. lactiflora* with and without “pockmarks” of the same age and diameter were compared (Figures 2A,B). The results showed that the contents of 11 compounds in the roots with “pockmarks” were higher than those in the samples without “pockmarks” (Table 2 and Supplementary Figure 6).

**FIGURE 6:**
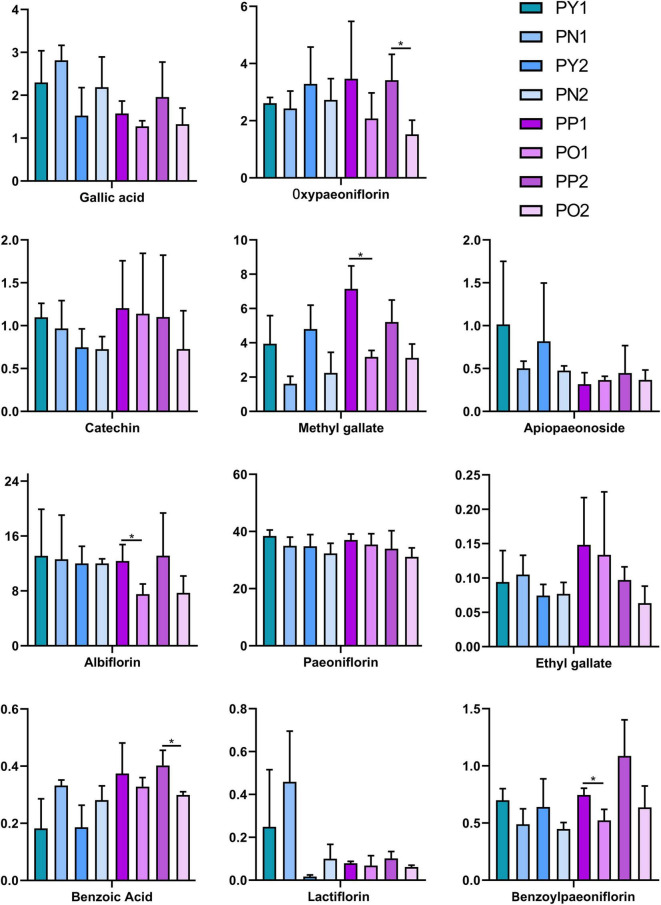
Contents of 11 reference compounds in the divided parts with and without “pockmarks” of *P. lactiflora* roots. *p < 0.05.

As shown in Figure 2, there are both old and newly formed “pockmarks” unevenly distributed on the surface of *P. lactiflora* roots, which are caused by the inconsistent time of being chewed by insects. The divided parts with and without “pockmarks” in the same root were further analyzed (Figures 2C,D). The results showed that the contents of paeoniflorin, albiflorin, oxypaeoniflorin, benzoylpaeoniflorin, methyl gallate, apiopaeonoside, and catechin were higher in the parts with “pockmarks,” while the contents of gallic acid, ethyl gallate, benzoic acid, and lactiflorin were higher in the parts without “pockmarks.” Furthermore, for the roots with “pockmarks” that were longitudinally cut (Figure 2E), there was a higher content of oxypaeoniflorin, catechin, methyl gallate, albiflorin, benzoylpaeoniflorin, gallic acid, ethyl gallate, benzoic acid, and paeoniflorin in the parts with “pockmarks” than that of parts without “pockmarks” (Figure 6).

### Correlation Analysis of Main Chemical Components in *P. lactiflora* Roots With Ages, Diameters and Pockmarks

In order to elucidate the relationship between the main active components in roots of *P. lactiflora* and their growth ages, diameters, and “pockmarks” characteristic, Pearson correlation analysis was performed on the 11 targeted compounds in *P. lactiflora* roots. The correlation analysis results were shown in Supplementary Figure 7. The correlations were color coded based on the coefficient values (warm and cold colors for positive and negative correlations, respectively), with the size of circles scaled according to their absolute values (Chang et al., 2020).

As shown in the correlation plot, there was a significant positive correlation between the diameter of the roots of *P. lactiflora* and its growth ages (*p* < 0.01). There was a significant negative correlation between the contents of oxypaeoniflorin, catechin, methyl gallate, and apiopaeonoside and growth ages of *P. lactiflora* roots (*p* < 0.05), while a significant positive correlation was observed between the content of lactiflorin and the growth ages (*p* < 0.0001). The contents of oxypaeoniflorin, albiflorin, methyl gallate, catechin, benzoic acid, and apiopaeonoside showed a significant negative correlation with the diameter of *P. lactiflora* roots (*p* < 0.01). The total contents of five monoterpene glycosides and the total contents of six phenols were also significantly negatively correlated with the root diameters (*p* < 0.0001). Regarding the characteristic of “pockmarks,” the contents of the other five phenols except benzoic acid were significantly positively correlated with “pockmarks” (*p* < 0.0001).

## Discussion

It is well known that the annual rings in the stems of most ligneous plants can be used to determine growth years (Schweingruber, 1988). In recent years, the growth rings have been widely accepted for determining the age of roots in perennial dicotyledonous herbs, such as *Salvia miltiorrhiza* (Wang et al., 2013), *Astragalus membranaceus* var. *mongholicus* (Peng et al., 2017b), and *P. lactiflora* (Chu et al., 2017). *P. lactiflora*, a perennial herb, is currently cultivated as an ornamental flower, and its roots can be used for medicinal purposes. In a previous field investigation, it was found that the local herbalists has cultivated *P. lactiflora* by radical bud propagation, so the root system of *P. lactiflora* was developed from adventitious roots produced annually. Consequently, the root system of *P. lactiflora* contains roots of different years. According to our previous study, the growth years of the *P. lactiflora* cultivated in Bozhou City is equal to the number of growth rings plus 1 (Zha et al., 2012), which was used to effectively identify the growth age of *P. lactiflora* roots. Many studies have shown that the quality of perennial Chinese herbal medicines is closely related to their growth years, such as *Panax ginseng* (Kim et al., 2012), *Centella asiatica* (Mohamad et al., 2018), and *Paris polyphylla* var. *yunnanensis* (Wang and Li, 2018), and their effective components differ depending on the growth years. Therefore, accurately determining the growth years of Chinese herbal medicines is the key to evaluate the quality of traditional Chinese medicines (Chen et al., 2021). Although various studies have determined the chemical constituents of *P. lactiflora* roots of different ages, the results are inconsistent, which might be due to the inability to determine the ages of *P. lactiflora* roots. Previous studies have considered the entire period of root cultivation as the actual growth period of *P. lactiflora* roots, but it is inaccurate because the root system of *P. lactiflora* contained roots of different years. Therefore, the age identification of *P. lactiflora* roots through the growth rings can more accurately evaluate the impact of growth years on the quality of *Shaoyao*. When a heat map was used for cluster analysis, the 3-, 4-, and 5-year-old samples of *P. lactiflora* each formed a cluster at an appropriate distance level. Consequently, this method can discriminate the samples from different years, which further confirms the reliability of using growth rings to identify the ages of *P. lactiflora*. After the accurate age identification of *P. lactiflora* roots, a unified processing method in this study was used to qualitatively and quantitatively analyze the roots of *P. lactiflora* of different ages. There were significant differences in the total content of five monoterpene glycosides (albiflorin, paeoniflorin, apiopaeonoside, benzoylpaeoniflorin, and oxypaeoniflorin) in *P. lactiflora* roots at different growth years, in which the content in 3-year-old roots was significantly higher than that in 4- and 5-year-old roots. Studies have reported that total glycosides of paeony (TGP) extracted from dried roots of *P. lactiflora* is the main active ingredient of *P. lactiflora* (Parker et al., 2016), which is mainly composed of paeoniflorin, oxypaeoniflorin, albiflorin, benzoylpaeoniflorin (Zhang and Wei, 2020). The 3-year-old *Shaoyao* contains higher levels of TGP, although its cultivation time was shorter, it might had better quality and medicinal value.

Generally, the thick roots of *P. lactiflora* are considered to be of higher quality and are prescribed as medicines, whereas the thin roots are industrially extracted (Wang et al., 2015). Although previous studies on *P. lactiflora* have found that root diameter affects the chemical component content of *P. lactiflora* roots, consistent results were not achieved might owing to the neglect of age difference in roots of different diameters (Wang et al., 2015; Li and Yuan, 2017). Therefore, the diameters of roots in the same age were considered to obtain accurate results in this study. The results showed that different diameters also had significant effects on the total content of five monoterpene glycosides and six phenols in *P. lactiflora* roots. The correlation plot showed strong negative correlations between the diameter and the contents of albiflorin, catechin, methyl gallate, and oxypaeoniflorin. In previous studies, paeoniflorin and its derivatives, such as oxypaeoniflorin and benzoylpaeoniflorin, were found to be more distributed in the periderm and cortex but less in the xylem (Li et al., 2016; Li B. et al., 2021). Previous studies have speculated that as the diameter increased, the proportion of xylem increased, whereas the proportion of cortex and phloem decreased. It has reported that the root cortex and phloem of *P. lactiflora* were rich in bioactive ingredients, especially the average content of paeoniflorin and albiflorin in the cortex of *P. lactiflora* was higher than that of phloem, vascular bundles of xylem and xylem rays (Wang et al., 2015). The results in this study showed that, the total content of five monoterpene glycosides and six phenols showed a downward trend with the increase in diameter of roots at the same age. Among them, the total content of 11 targeted chemical compounds were higher in 3- and 4-year-old roots with a diameter of 0.7–1 cm, and 5-year-old roots with a diameter of 1–1.5 cm. The thin roots have a larger proportion of the cortex and phloem with a higher content of main chemical components, which is consistent with the reported studies.

The cultivation of *P. lactiflora* in Bozhou City has a long history, which can be traced back 1000 years ago. During the cultivation process, *P. lactiflora* often suffer damage caused by insects, such as *Holotrichia diomphalia* bates and *Gryllotalpa unispina* Saussure. When herbivorous insects feed, they physically lacerate tissue and cause wounds, which will form “pockmarks” on the root surface of *P. lactiflora*. *Shaoyao* with solid textures and smooth surfaces is often regarded as excellent products, and roots with “pockmarks” tend to lower its market prices. Therefore, to get a better appearance, pesticides are used to prevent “pockmarks” caused by insects in planted *P. lactiflora*. However, overuse of pesticides increases planting costs, causing the harmful substances in *P. lactiflora* roots to exceed the standards, which in turn affects the quality of *Shaoyao*. Previous studies have found that different morphological characters can affect the quality of medicinal materials (Chen et al., 2014; Chu et al., 2020; Zhao et al., 2021). The “pockmarks” is a special morphological character of *Shaoyao*, how it impacts on the quality of *Shaoyao* needs further investigation. Therefore, *P. lactiflora* roots with and without “pockmarks” were compared in our study. The results of multivariate statistical analysis showed that there were significant differences in metabolite levels between *P. lactiflora* roots with “pockmarks” and those without “pockmarks.” The results of quantitative experiments showed that there was a higher content of 11 targeted chemicals in samples with “pockmarks” than in those without “pockmarks.” It is speculated that *Shaoyao* with “pockmarks” might have better medicinal value for clinical practice. Thus, it is necessary to comprehensively evaluate the quality of medicinal materials, instead of focusing on appearance aesthetics.

During feeding by herbivorous insects, plants are subjected to a multitude of stimuli, resulting in complex and cumulative defense responses (Waterman et al., 2019). Among these defenses, secondary metabolites function as antiherbivore defenses (Agrawal and Weber, 2015). For example, a large increase in secondary metabolites may occur in stressed plants subjected to mechanical or insect damage (Harborne, 1990). Several plant species, such as *Medicago truncatula* (Leitner et al., 2005), *Zea mays* (Degenhardt, 2009), and *Helianthus annuus* (Mason et al., 2016), release terpenoids and phenolic compounds after damage by herbivores. Consequently, after the roots are often attacked by insects, there is an increase in the content of monoterpene glycosides and phenols, which might be related to the defense mechanism of *P. lactiflora*. Considering the role of secondary metabolites in ecological and evolutionary processes, previous studies on *P. lactiflora* have suggested that the accumulation of gallotannins in the cork and xylem of *P. lactiflora* roots may be related to the ability to form a chemical barrier against enemies (Li et al., 2016). In this study, 20 potential difference markers of *P. lactiflora* roots with and without “pockmarks” were screened. Among them, three tannins, six monoterpene glycosides, and a phenolic were identifiable. Additionally, the contents of methyl gallate, albiflorin, and benzoylpaeoniflorin were significantly different in these two parts. These compounds increased in the damaged parts of *P. lactiflora* roots, which might be related to its defensive chemical reactions, but the specific reaction mechanism still needs further study.

## Conclusion

In the present study, an efficient qualitative UPLC-Q/TOF-MS and quantitative HPLC-DAD method were established to systematically clarify the compositional differences of the main active ingredients of *P. lactiflora* roots with different ages, diameters, and “pockmarks” characteristics. Qualitative results showed that the chemical compositions of different *P. lactiflora* samples were similar, and a total of 60 specialized metabolites were identified and characterized. Furthermore, 20 potential quality markers were first discovered in *P. lactiflora* roots with and without “pockmarks.” Quantitative analysis results showed that the quality of 3-year-old *Shaoyao* maybe better when compared with 4- and 5-year-old ones. For *P. lactiflora* samples in the same age, the total contents of five monoterpene glycosides and six phenols were increased with a decrease in diameter. Furthermore, the roots with “pockmarks” had higher contents of 11 targeted compounds, showing its important medicinal value. This study comprehensively compared the chemical characteristics of *Shaoyao* with different ages, diameters, and “pockmarks,” which provided a basis for further quality evaluation and rational utilization of *P. lactiflora* resources.

## Data Availability Statement

The original contributions presented in the study are included in the article/Supplementary Material, further inquiries can be directed to the corresponding author/s.

## Author Contributions

XZ, LH, and HP conceived and designed the experiments. XZ, SC, MY, and ZY assisted and performed the experiments. YZ provided the samples. XZ wrote the manuscript. XZ, SC, and MZY conducted the data analyses. All authors contributed to the article and approved the submitted version.

## Conflict of Interest

The authors declare that the research was conducted in the absence of any commercial or financial relationships that could be construed as a potential conflict of interest.

## Publisher’s Note

All claims expressed in this article are solely those of the authors and do not necessarily represent those of their affiliated organizations, or those of the publisher, the editors and the reviewers. Any product that may be evaluated in this article, or claim that may be made by its manufacturer, is not guaranteed or endorsed by the publisher.
