# Supplementary material for: Comparative Elucidation of Age, Diameter, and “Pockmarks” in Roots of Paeonia lactiflora Pall. (Shaoyao) by Qualitative and Quantitative Methods
Source: Front Plant Sci. 2022 Jan 26;12:802196. doi: 10.3389/fpls.2021.802196 (PMC8826210; doi:10.3389/fpls.2021.802196)
Supplement: Supplementary file 1 [file Data_Sheet_1.docx]

**Supplementary Material**

# Supplementary Figures

Supplementary Figure 1. The structures of the 14 reference compounds. Paeoniflorin (A); oxypaeoniflorin (B); benzoylpaeoniflorin (C); albiflorin (D); apiopaeonoside (E); lactiflorin (F); 1,2,3,4,6-O-pentagalloylglucose (G); 3,4-Dihydroxybenzaldehyde (H); 3,4-Dihydroxybenzoic acid (I); benzoic acid (J); methyl gallate (K); catechin (L); ethyl gallate (M); gallic acid (N).

Supplementary Figure 2. The MS data of the 14 reference compounds.

Supplementary Figure 3. HPLC-DAD chromatogram of 11 reference compounds (A) and sample of *P. lactiflora* (B). Gallic acid (1); oxypaeoniflorin (2); catechin (3); methyl gallate (4); apiopaeonoside (5); albiflorin (6); paeoniflorin (7); ethyl gallate (8); benzoic acid (9); lactiflorin (10); benzoylpaeoniflorin (11).

Supplementary Figure 4. The TIC chromatogram of *P. lactiflora* roots with and without “pockmarks” in negative mode.

Supplementary Figure 5. PCA score plot (A) and permutation plot (after 200 times) of OPLS-DA score plot (B) of the with and without “pockmarks” of *P. lactiflora* roots; PCA score plot (C) and permutation plot (after 200 times) of OPLS-DA score plot (D) of the divided parts with and without “pockmarks” of *P. lactiflora* roots; S-plot (E) and Plot List (F) of the potential quality markers for the divided parts with and without “pockmarks” of *P. lactiflora* roots. The blue dot represents the whole root with “pockmarks”, green dot represents the whole root without “pockmarks”, red represents QC samples, orange dot represents divided root without “pockmarks”, and grey dot represents divided root with “pockmarks”.

Supplementary Figure 6. The contents of 11 reference compounds in the whole roots of *P. lactiflora* with and without “pockmarks” of the same age and diameter.

Supplementary Figure 7. Correlation plot of the content of 11 reference compounds in *P. lactiflora* roots with ages, diameters, and pockmarks. TMG, the total content of five monoterpene glycosides including oxypaeoniflorin, albiflorin, paeoniflorin, lactiflorin and benzoylpaeoniflorin; TPC, the total content of six phenols including gallic acid, catechin, methyl gallate, ethyl gallate, apiopaeonoside and benzoic acid.

**Supplementary Tables**

Supplementary Table 1. Collection information of *P. lactiflora* roots samples with different diameters and “pockmarks”.

Supplementary Table 2. Characterization of the specialized metabolites in *P. lactiflora* roots using UPLC-Q/TOF-MS in ESI^−^.

Supplementary Table 3. Potential quality markers for the divided parts with and without “pockmarks” of *P. lactiflora* roots.


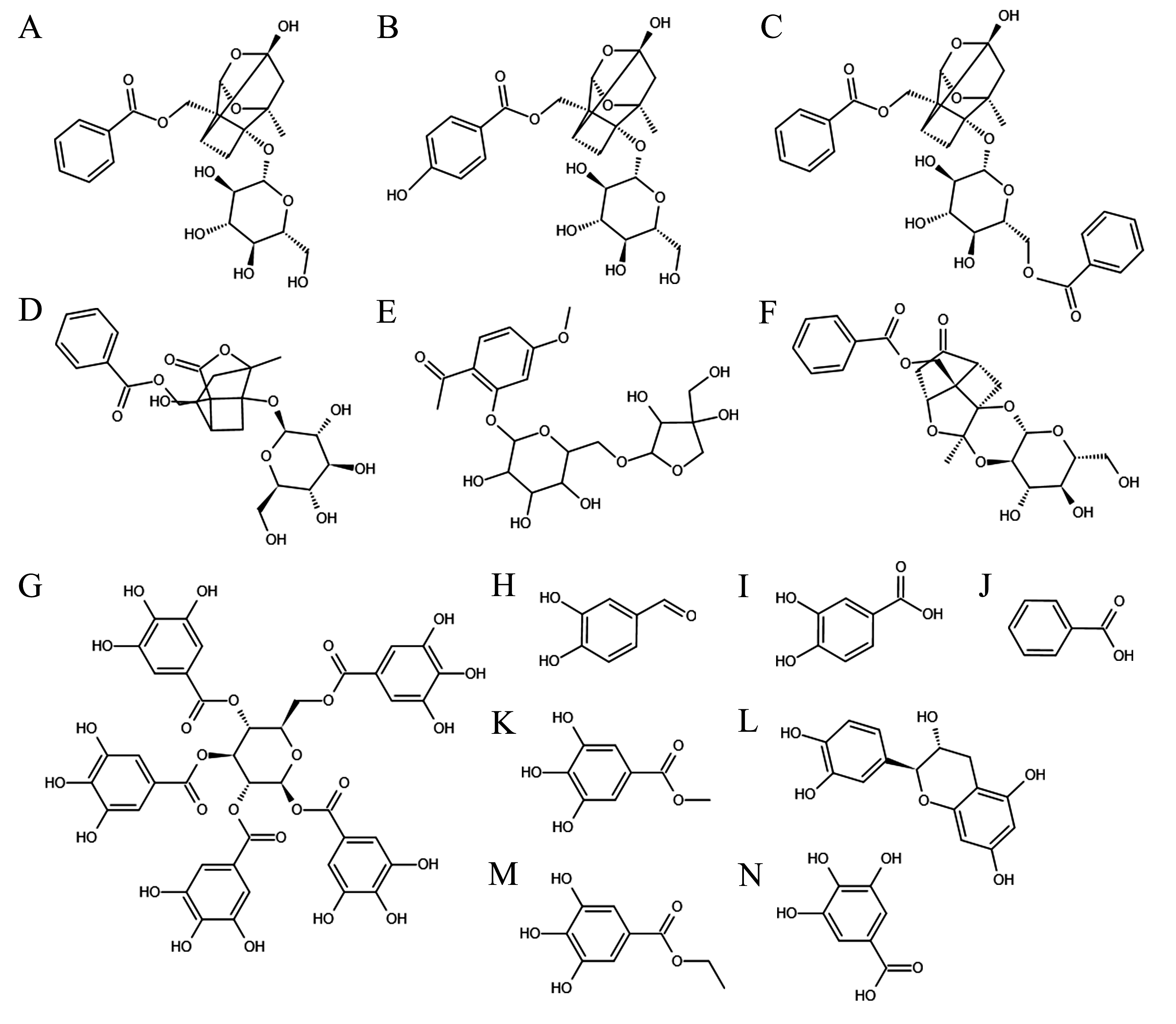


Supplementary Figure 1. The structures of the 14 reference compounds. Paeoniflorin (A); oxypaeoniflorin (B); benzoylpaeoniflorin (C); albiflorin (D); apiopaeonoside (E); lactiflorin (F); 1,2,3,4,6-O-pentagalloylglucose (G); 3,4-Dihydroxybenzaldehyde (H); 3,4-Dihydroxybenzoic acid (I); benzoic acid (J); methyl gallate (K); catechin (L); ethyl gallate (M); gallic acid (N).

Gallic acid

3, 4-Dihydroxybenzoic acid

3, 4-Dihydroxybenzaldehyde

Oxypaeoniflorin

Catechin

Methy gallate

Apiopaeonoside

Albiflorin

Paeoniflorin

Ethyl gallate

1,2,3,4,6-0-pentagalloylglucose

Benzoic acid

Lactiflorin

Benzoylpaeoniflorin

Supplementary Figure 2. The MS data of the 14 reference compounds.


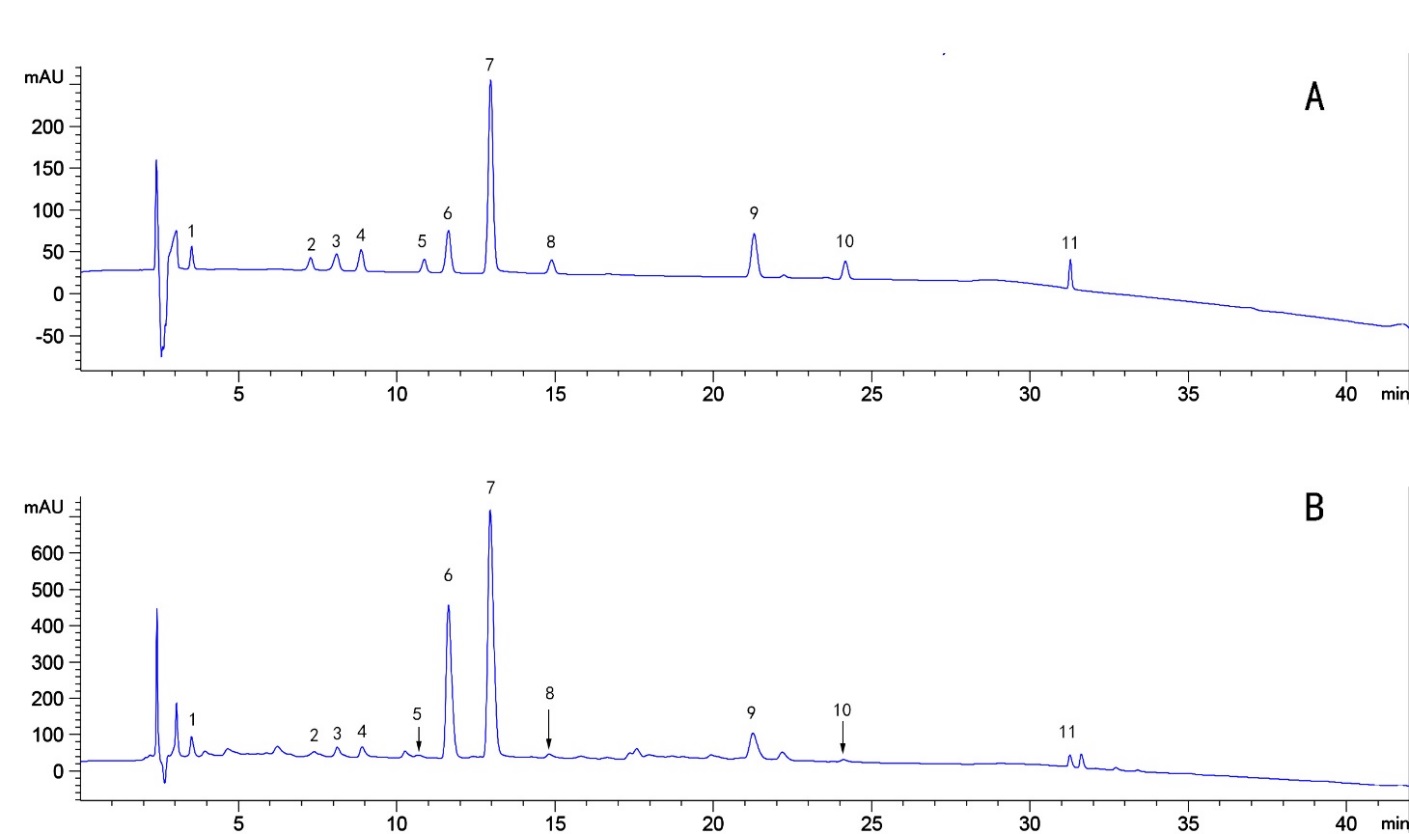


Supplementary Figure 3. HPLC-DAD chromatogram of 11 reference compounds (A) and sample of *P. lactiflora* roots (B). Gallic acid (1); oxypaeoniflorin (2); catechin (3); methyl gallate (4); apiopaeonoside (5); albiflorin (6); paeoniflorin (7);

ethyl gallate (8); benzoic acid (9); lactiflorin (10); benzoylpaeoniflorin (11).


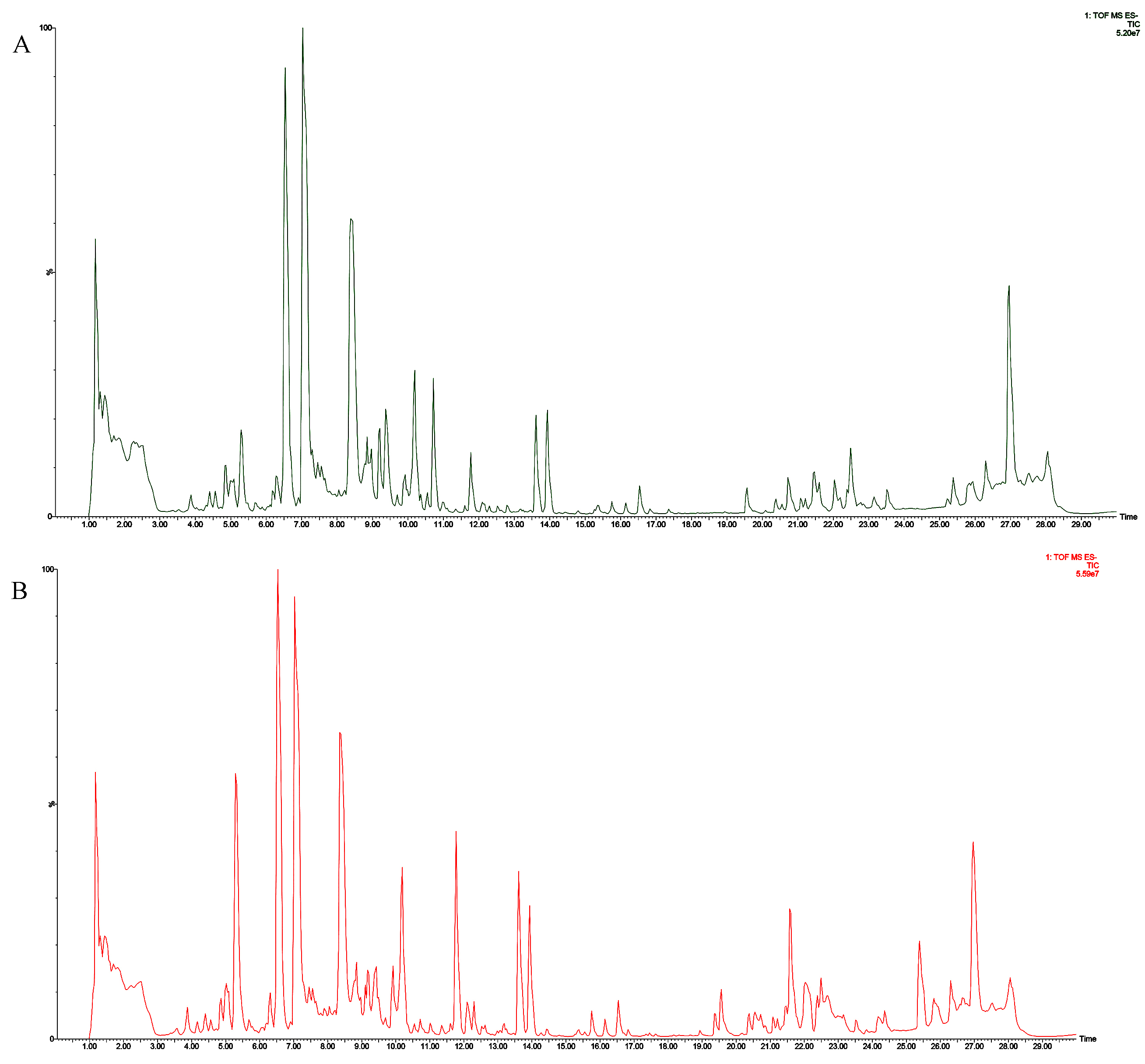


Supplementary Figure 4. The TIC chromatogram of *P. lactiflora* roots with and without “pockmarks” in negative mode.


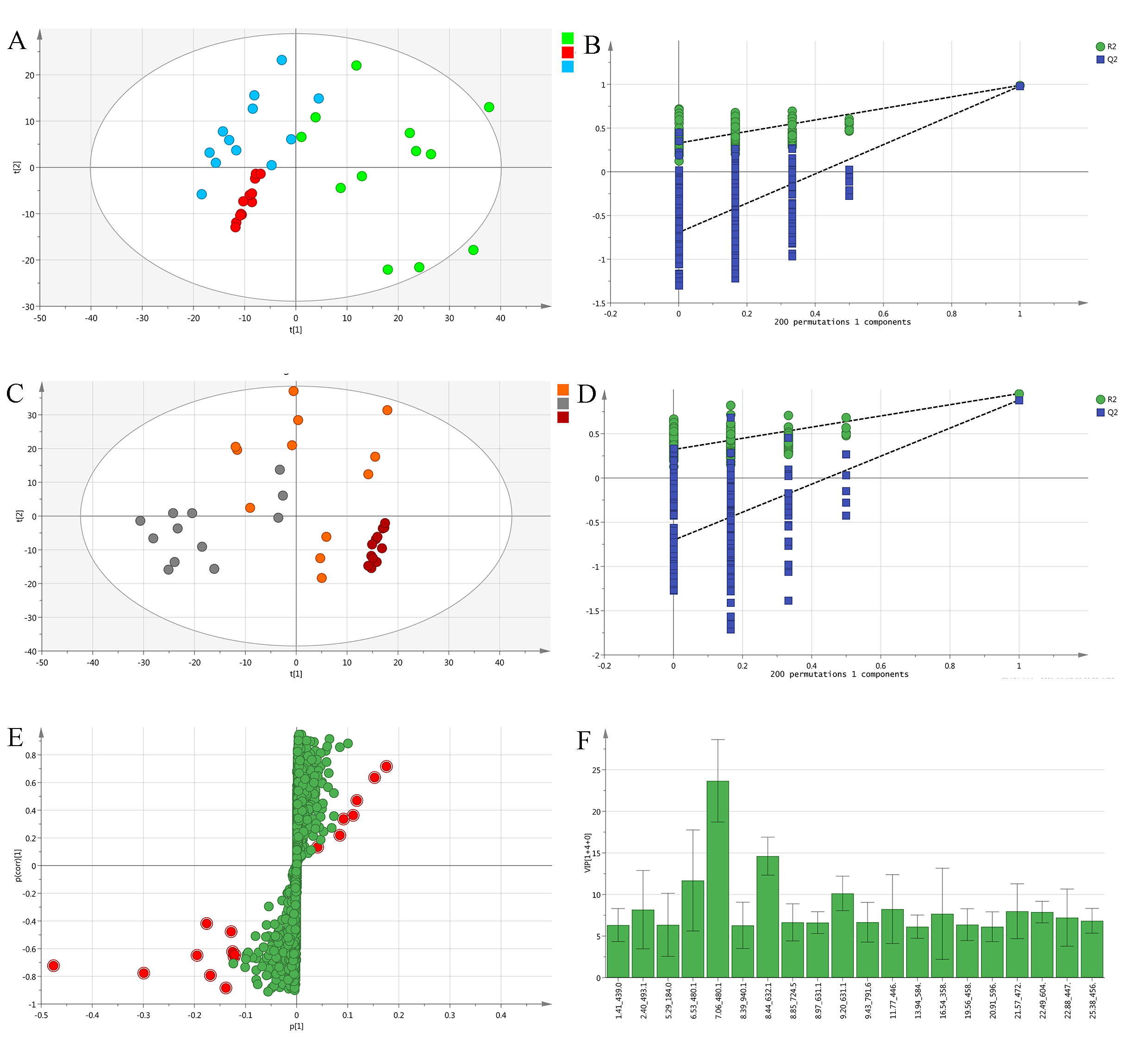


Supplementary Figure 5. PCA score plot (A) and permutation plot (after 200 times) of OPLS-DA score plot (B) of the with and without “pockmarks” of *P. lactiflora* roots; PCA score plot (C) and permutation plot (after 200 times) of OPLS-DA score plot (D) of the divided parts with and without “pockmarks” of *P. lactiflora* roots; S-plot (E) and Plot List (F) of the potential quality markers for the divided parts with and without “pockmarks” of *P. lactiflora* roots. The blue dot represents the whole root with “pockmarks”, green dot represents the whole root without “pockmarks”, red represents QC samples, orange dot represents divided root without “pockmarks”, and grey dot represents divided root with “pockmarks”.


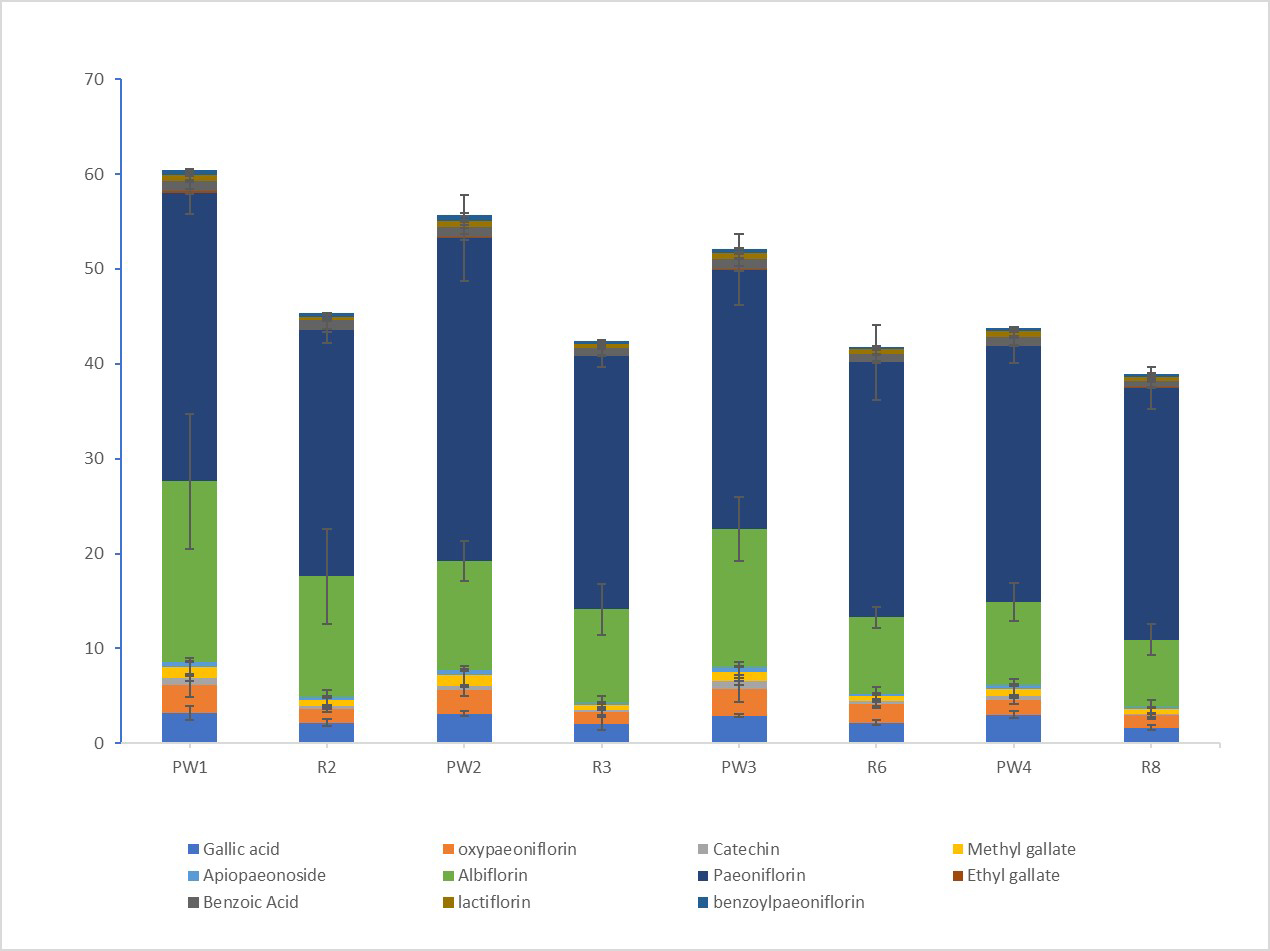


Supplementary Figure 6. The contents of 11 reference compounds in the whole roots of *P. lactiflora* with and without “pockmarks” of the same age and diameter.


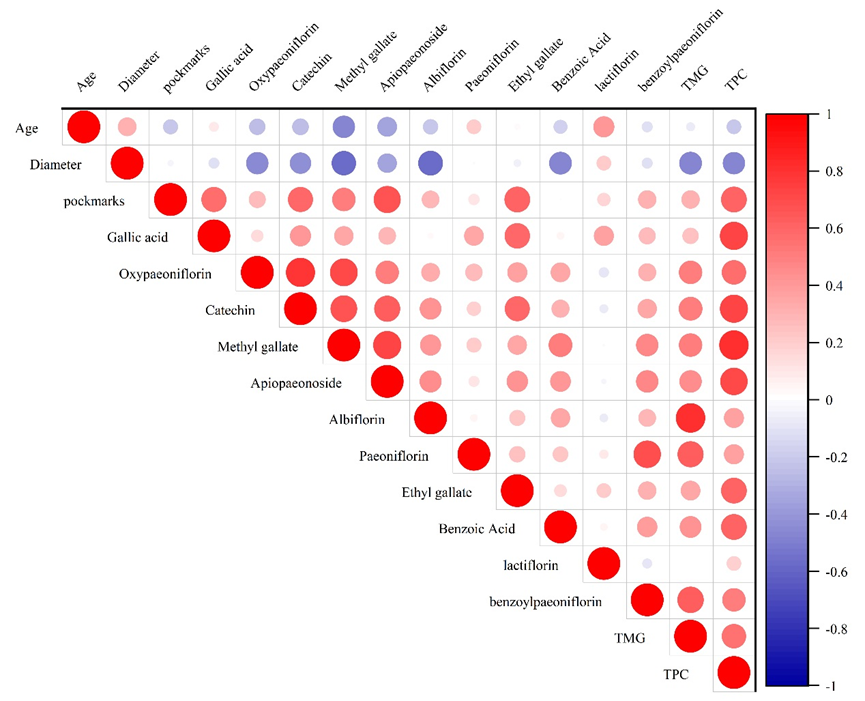


Supplementary Figure 7. Correlation plot of the content of 11 reference compounds in *P. lactiflora* roots with ages, diameters, and pockmarks. TMG, the total content of five monoterpene glycosides including oxypaeoniflorin, albiflorin, paeoniflorin, lactiflorin and benzoylpaeoniflorin; TPC, the total content of six phenols including gallic acid, catechin, methyl gallate, ethyl gallate, apiopaeonoside and benzoic acid.

Supplementary Table 1. Collection information of *P. lactiflora* roots samples with different diameters and “pockmarks”.

| No. | Sample name | Sample size | Ages | Middle diameter (cm) | Distribution of “pockmark” |
| --- | --- | --- | --- | --- | --- |
| 1 | R1 | 7 | 3 | 0.7-1 | Almost no distribution |
| 2 | R2 | 7 | 3 | 1.1-1.5 | Almost no distribution |
| 3 | R3 | 8 | 3 | 1.6-2 | Almost no distribution |
| 4 | R4 | 4 | 3 | 2-2.5 | Almost no distribution |
| 5 | R5 | 7 | 4 | 0.7-1 | Almost no distribution |
| 6 | R6 | 7 | 4 | 1-1.5 | Almost no distribution |
| 7 | R7 | 8 | 4 | 1.6-2 | Almost no distribution |
| 8 | R8 | 7 | 4 | 2-2.8 | Almost no distribution |
| 9 | R9 | 7 | 5 | 1-1.5 | Almost no distribution |
| 10 | R10 | 7 | 5 | 1.6-2 | Almost no distribution |
| 11 | R11 | 7 | 5 | 2-2.8 | Almost no distribution |
| 12 | PW1 | 3 | 3 | 1-1.5 | Wide distribution |
| 13 | PW2 | 3 | 3 | 1.6-2 | Wide distribution |
| 14 | PW3 | 3 | 4 | 1-1.5 | Wide distribution |
| 15 | PW4 | 3 | 4 | 2-2.8 | Wide distribution |
| 16 | PY1 | 3 | 5 | 1.6-2 | Partial distribution |
|  | PN1 | 3 | 5 | 1.6-2 | Almost no distribution |
| 17 | PY2 | 3 | 4 | 1.6-2 | Partial distribution |
|  | PN2 | 3 | 4 | 1.6-2 | Almost no distribution |
| 18 | PP1 | 3 | 5 | 1.6-2 | Partial distribution |
|  | PO1 | 3 | 5 | 1.6-2 | Almost no distribution |
| 19 | PP2 | 3 | 5 | 2-2.8 | Partial distribution |
|  | PO2 | 3 | 5 | 2-2.8 | Almost no distribution |

R. Samples of different diameters; PW. whole roots with “pockmarks”; PY1. divided roots with old formed “pockmarks”; PN1. divided roots without old formed “pockmarks”; PY2. divided roots with newly formed “pockmarks”; PN2. divided roots without newly formed “pockmarks”; PP. parts with “pockmarks” of the longitudinally cut root; PO. parts without “pockmarks” of the longitudinally cut root.

Supplementary Table 2. Characterization of the specialized metabolites in *P. lactiflora* roots using UPLC-Q/TOF-MS in ESI^−^.

| No. | t_R_(min) | Molecular formula | Adduct ion | Theoretical | Experimental | Error(ppm) | MS/MS | Identefication | References |
| --- | --- | --- | --- | --- | --- | --- | --- | --- | --- |
| 1 | 1.19 | C_12_H_22_0_11_ | [M−H] ^–^ | 341.1084 | 341.11 | 4.69 | 179.0595,143.0396,119.0378,113.0271,101.0262,89.0254 | Sucrose | Li et al., 2021 |
|  |  |  | [M−H+HCOOH]^–^ | 387.1139 | 387.1168 | 7.49 |  |  |  |
| 2 | 1.6 | C_6_H_8_O_7_ | [M−H] ^–^ | 191.0192 | 191.0193 | 0.52 | 191.0221,111.0108,87.0096,85.0301 | Citric acid | Tan et al., 2017 |
| 3 | 1.72 | C_13_H_16_O_10_ | [M−H] ^–^ | 331.0665 | 331.0675 | 3.02 | 271.0546,211.0297,169.0180 | Glucogallin | Li et al., 2021 |
| 4^*^ | 2.26 | C_7_H_6_O_5_ | [M−H] ^–^ | 169.0137 | 169.0153 | 9.47 | 125.0256 | Gallic acid | Li et al., 2021 |
| 5 | 2.37 | C_19_H_26_O_15_ | [M−H] ^–^ | 493.1193 | 493.1204 | 2.23 | 331.0786,169.0180,125.0256 | Galloylsucrose or isomer | Liu et al.,2015 |
| 6 | 2.43 | C_16_H_24_O_9_ | [M−H] ^–^ | 359.1342 | 359.1356 | 3.9 | 197.0852,179.0623,161.0479 | 1-O-β-D-glucopyransoyl-paeonisuffron or isomer | Wang et al., 2015 |
|  |  |  | [M−H+HCOOH]^–^ | 405.1397 | 405.1421 | 5.92 |  |  |  |
| 7 | 3.45 | C_19_H_26_O_15_ | [M−H] ^–^ | 493.1193 | 493.1214 | 4.26 | 331.0823,169.0206 | Galloylsucrose or isomer | Liu et al., 2015 |
| 8 | 3.54 | C_23_H_28_O_14_ | [M−H] ^–^ | 527.1401 | 527.1421 | 3.79 | 497.1366,313.0560,169.0153,125.9256 | Galloyl-desbenzoylpaeoniflorin | Li et al., 2021 |
| 9 | 3.57 | C_34_H_24_O_22_ | [M−H] ^–^ | 783.0681 | 783.0752 | 9.07 | 481.08,301.0052,169.018,125.0256 | Pedunculagin or isomer | Zhan et al., 2018 |
| 10 | 3.88 | C_16_H_24_O_9_ | [M−H] ^–^ | 359.1342 | 359.1356 | 3.9 | 241.9003,275.0407 | 6-O-β-D-glucopyranosyl lactinolide | Liu et al., 2015 |
|  |  |  | [M−H+HCOOH]^–^ | 405.1397 | 405.1421 | 5.92 |  |  |  |
| 11^*^ | 3.91 | C_7_H_6_O_4_ | [M−H] ^–^ | 153.0188 | 153.0196 | 5.23 | 109.0339 | 3,4-Dihydroxybenzoic acid | Liu et al., 2015 |
| 12 | 4.16 | C_16_H_24_O_8_ | [M−H] ^–^ | 343.1393 | 343.1425 | 9.33 | 181.0889,151.0766 | Mudanpioside F | Liu et al., 2015 |
| 13 | 4.37 | C_34_H_24_O_22_ | [M−H] ^–^ | 783.0681 | 783.0752 | 9.07 | 301.0553,169.0180,125.0256 | Pedunculagin or isomer | Zhan et al., 2018 |
| 14^*^ | 4.87 | C_23_H_28_O_12_ | [M−H] ^–^ | 495.1503 | 495.1538 | 7.07 | 465.1458,137.0272 | Oxypaeoniflorin | Li et al., 2021 |
|  |  |  | [M−H+HCOOH]^–^ | 541.1557 | 541.1554 | -0.55 |  |  |  |
| 15 | 4.91 | C_27_H_24_O_18_ | [M−H] ^–^ | 635.0884 | 635.0879 | -0.79 | 465.1547,313.0597,169.0180 | Trigalloyl glucose | Li et al., 2021 |
| 16 | 4.93 | C_45_H_38_O_18_ | [M−H] ^–^ | 865.198 | 865.2061 | 9.36 | 635.1034,289.0909,137.0272,125.0256 | Procyanidin C1 or isomers | Zhan et al., 2018 |
| 17^*^ | 5.02 | C_7_H_6_O_3_ | [M−H] ^–^ | 137.0239 | 137.0248 | 6.57 | 109.0296 | 3,4-Dihydroxybenzaldehyde | Liu et al., 2015 |
| 18^*^ | 5.09 | C_15_H_14_O_6_ | [M−H] ^–^ | 289.0712 | 289.0725 | 4.5 | 245.0860,205.0558,179.0376,165.0220,137.0279,109.0296 | Catechin | Li et al., 2021 |
| 19 | 5.27 | C_24_H_30_O_13_ | [M−H] ^–^ | 525.1608 | 525.166 | 9.9 | 495.1629,429.1254,295.0456 | Mudanpioside E | Liu et al., 2015 |
| 20^*^ | 5.29 | C_8_H_8_O_5_ | [M−H] ^–^ | 183.0293 | 183.0299 | 3.28 | 168.0085,124.0185 | Methyl gallate | Li et al., 2021 |
| 21 | 5.68 | C_15_H_20_O_8_ | [M−H] ^–^ | 327.108 | 327.1065 | -4.59 | 205.0383,161.0479,143.0347,121.0309 | Paeonoside | Li et al., 2017 |
| 22 | 5.85 | C_27_H_24_O_18_ | [M−H] ^–^ | 635.0884 | 635.0879 | -0.79 | 465.0708,313.0597,169.0180 | Trigalloyl glucose | Li et al., 2021 |
| 23 | 6.29 | C_19_H_25_O_12_ | [M−H] ^–^ | 445.1346 | 445.139 | 9.88 | 323.1031， | 1'-O-Benzoylsucrose |  |
| 24^*^ | 6.3 | C_20_H_28_O_12_ | [M−H] ^–^ | 459.1503 | 459.1539 | 7.84 | 293.0909,165.0588,150.0346 | Apiopaeonoside | Zhan et al., 2018 |
|  |  |  | [M−H+HCOOH]^–^ | 505.1557 | 505.1579 | 4.36 |  |  |  |
| 25^*^ | 6.53 | C_23_H_28_O_11_ | [M−H] ^–^ | 479.1553 | 479.1542 | -2.3 | 357.1258,283.0847,121.0293 | Albiflorin | Liu et al., 2015 |
|  |  |  | [M−H+HCOOH]^–^ | 525.1608 | 525.1614 | 1.14 |  |  |  |
| 26^*^ | 7.03 | C_23_H_28_O_11_ | [M−H] ^–^ | 479.1553 | 479.1546 | -1.46 | 449.1503,357.1258,327.1139,195.0679，165.0588,121.0309 | Paeoniflorin | Li et al., 2021 |
|  |  |  | [M−H+HCOOH]^–^ | 525.1608 | 525.1614 | 1.14 |  |  |  |
| 27 | 7.3 | C_34_H_28_O_22_ | [M−H] ^–^ | 787.0994 | 787.1058 | 8.13 | 635.1034,169.0180 | Tetragalloyl glucose | Li et al., 2021 |
| 28 | 7.56 | C_34_H_28_O_22_ | [M−H] ^–^ | 787.0994 | 787.1058 | 8.13 | 635.0982,617.0904,465.0752,313.0597, 295.0456,121.0309 | Tetragalloyl glucose | Li et al., 2021 |
| 29^*^ | 7.67 | C_9_H_10_O_5_ | [M−H] ^–^ | 197.045 | 197.045 | 0 | 169.0153 | Ethyl gallate | Liu et al., 2015 |
| 30 | 8.04 | C_27_H_32_O_16_ | [M−H] ^–^ | 611.1612 | 611.1571 | -6.71 | 301.0016,169.0153,125.0256, | Suffruticoside A or isomer | Li et al., 2021 |
| 31^*^ | 8.39 | C_41_H_32_O_26_ | [M−H] ^–^ | 939.1104 | 939.111 | 0.64 | 769.1083,631.1797,469.0555,169.0153 | 1,2,3,4,6-O-Pentagalloylglucose | Wang et al., 2015 |
| 32 | 8.44 | C_30_H_32_O_15_ | [M−H] ^–^ | 631.1663 | 631.1694 | 4.91 | 613.1672,491.1231,399.0976,313.0597,211.0267,169.0153,125.0256 | Galloylalbiflorin or Galloylpaeoniflorin | Wang et al., 2015 |
| 33 | 8.8 | C_30_H_32_O_16_ | [M−H] ^–^ | 647.1612 | 647.1658 | 7.11 | 509.2181,331.1757,271.0479,211.0297,169.0153,165.0562 | Galloyloxypaeoniflorin | Wang et al., 2015 |
| 34 | 8.93 | C_30_H_32_O_15_ | [M−H] ^–^ | 631.1663 | 631.1694 | 4.91 | 613.1722,313.059,125.0256,121.0309 | Galloylalbiflorin or Galloylpaeoniflorin | Wang et al., 2015 |
| 35 | 9.1 | C_48_H_36_O_30_ | [M−H] ^–^ | 1091.121 | 1091.129 | 7.24 | 939.1392,769.1083,617.0955,545.0612,469.0555,169.0180,125.0240 | Hexagalloyl glucose | Li et al., 2021 |
| 36 | 9.19 | C_30_H_32_O_15_ | [M−H] ^–^ | 631.1663 | 631.1692 | 4.59 | 587.1354,525.1474,479.1699 | Galloylalbiflorin or Galloylpaeoniflorin | Wang et al., 2015 |
| 37 | 9.34 | C_48_H_36_O_30_ | [M−H] ^–^ | 1091.121 | 1091.129 | 7.24 | 939.1392,769.1083,617.0955,545.0659,469.0599,447.0624,169.0153,125.0256 | Hexagalloyl glucose | Li et al., 2021 |
| 38 | 9.37 | C_30_H_32_O_15_ | [M−H] ^–^ | 631.1663 | 631.1694 | 4.91 | 3130596,169.0153,125.0256 | Galloylalbiflorin or Galloylpaeoniflorin | Wang et al., 2015 |
| 39^*^ | 9.87 | C_7_H_6_O_2_ | [M−H] ^–^ | 121.029 | 121.0287 | -2.48 | 103.6157 | Benzoic acid |  |
| 40 | 9.92 | C_22_H_18_O_11_ | [M−H+HCOOH]^–^ | 503.0826 | 503.0812 | -2.78 | 457.1743,163.0798 | (-)-Gallocatechin gallate | Tan et al., 2017 |
| 41 | 10.19 | C_23_H_28_O_11_ | [M−H] ^–^ | 479.1632 | 479.1654 | 4.59 | 449.1416,327.1102,165,121.0309 | Iso-apiopaeonoside | Li et al., 2021 |
|  |  |  | [M−H+HCOOH]^–^ | 525.168 | 525.1661 | -3.62 |  |  |  |
|  |  |  | [2M−H] ^–^ | 959.3185 | 959.314 | -4.69 |  |  |  |
| 42 | 10.62 | C_7_H_6_O_3_ | [M−H+HCOOH]^–^ | 137.0239 | 137.0248 | 6.57 | 121.0309 | P-Hydroxybenzoic acid | Hu et al., 2016 |
| 43^*^ | 10.72 | C_23_H_26_O_10_ | [M−H] ^–^ | 461.1448 | 461.1427 | -4.55 | 339.1098,283.0879,177.0580 | lactiflorin | Shi et al., 2016 |
|  |  |  | [M−H+HCOOH]^–^ | 507.1503 | 507.1515 | 2.37 |  |  |  |
| 44 | 11.01 | C_20_H_28_O_12_ | [M−H] ^–^ | 459.1503 | 459.1542 | 8.49 | 293.098,233.0665,165.0562 | Paeonolide or isomer | Li et al., 2017 |
|  |  |  | [M−H+HCOOH]^–^ | 505.1557 | 505.1579 | 4.36 |  |  |  |
| 45 | 11.62 | C_25_H_32_O_11_ | [M−H]^–^ | 507.1866 | 507.1883 | 3.35 | 461.2394,121.0309 | 4-O-Ethyl paeoniflorin | Li et al., 2017 |
| 46 | 11.77 | C_19_H_26_O_12_ | [M−H]^–^ | 445.1346 | 445.1347 | 0.22 | 411.0743,323.1031,283.0916 | benzoylsucrose | Li et al., 2016 |
|  |  |  | [M−H+HCOOH]^–^ | 491.1401 | 491.1412 | 2.24 |  |  |  |
| 47 | 11.82 | C_25_H_30_O_12_ | [M−H] ^–^ | 521.1659 | 521.1642 | -3.26 | 491.2228,121.0309 | Acetylpaeoniflorin | Li et al., 2017 |
| 48 | 12.8 | C_31_H_34_O_13_ | [M−H] ^–^ | 613.1921 | 613.1925 | 0.65 | 461.2438,321.1382,151.0062,121.0309 | Mudanpioside A or isomer | Li et al., 2017 |
| 49^*^ | 13.61 | C_30_H_32_O_12_ | [M−H] ^–^ | 583.1816 | 583.1852 | 6.17 | 553.1843,431.1414,165.0588,121.0309 | Benzoylpaeoniflorin | Li et al., 2021 |
| 50 | 13.94 | C_30_H_32_O_12_ | [M−H] ^–^ | 583.1816 | 583.1852 | 6.17 | 553.1843,431.1414,165.0588,121.0309 | Iso-benzoyloxypaeoniflorin | Li et al., 2021 |
|  |  |  | [M−H+HCOOH]^–^ | 629.187 | 629.1918 | 7.63 |  |  |  |
| 51 | 14.43 | C_10_H_12_O_4_ | [M−H] ^–^ | 195.0657 | 195.0651 | -3.08 | 177.0580,137.0224,123.0519,87.0115 | Paeonilactone B or paeonilactone C | Li et al., 2021 |
| 52 | 15.55 | C_31_H_34_O_14_ | [M−H] ^–^ | 629.187 | 629.1866 | -0.64 | 599.1862 | Mudanpioside J or isomer | Li et al., 2021 |
| 53 | 16.15 | C_31_H_34_O_14_ | [M−H] ^–^ | 629.187 | 629.1866 | -0.64 | 599.1862 | Mudanpioside J or isomer | Li et al., 2021 |
| 54 | 16.57 | C_22_H_30_O_4_ | [M−H] ^–^ | 357.2066 | 357.207 | 1.12 | 325.1811 | Palbinone isomer | Li et al., 2017 |
| 55 | 17.31 | C_24_H_30_O_12_ | [M−H] ^–^ | 509.1659 | 509.1673 | 2.75 | 479.2102,461.1900,357.2147 | Mudanpioside D |  |
| 56 | 20.55 | C_30_H_48_O_3_ | [M−H] ^–^ | 455.3525 | 455.356 | 7.69 | 437.2859,391.0321 | Oleanolic acid or isomer | Li et al., 2017 |
| 57 | 21.36 | C_24_H_30_O_13_ | [M−H] ^–^ | 525.1608 | 525.1614 | 1.14 | 373.2164,343.1956,125.8713 | Isomer of mudanpioside E | Wang et al., 2015 |
|  |  |  | [M−H+HCOOH]^–^ | 571.1663 | 571.1651 | -2.1 |  |  |  |
| 58 | 22.03 | C_30_H_48_O_4_ | [M−H] ^–^ | 471.3474 | 471.3496 | 4.67 | 393.3217 | Hederagenin or isomer | Wang et al., 2015 |
|  |  |  | [M−H+HCOOH]^–^ | 517.3529 | 517.3499 | -5.8 |  |  |  |
| 59 | 25.4 | C_30_H_48_O_3_ | [M−H] ^–^ | 455.3525 | 455.356 | 7.69 | 437.2774,391.2345 | Oleanolic acid or isomer | Li et al., 2017 |
| 60 | 25.78 | C_16_H_22_O_4_ | [M−H] ^–^ | 277.144 | 277.1426 | -5.05 | 134.8964 | Dibutyl phthalate | Wang et al., 2015 |

^*^Identified by comparing with standard compounds.

Supplementary Table 3. Potential quality markers for the divided parts with and without “pockmarks” of *P. lactiflora* roots.

| NO. | Identification | Molecularformula | t_R_(min) | [M−H] ^–^ | VIP |
| --- | --- | --- | --- | --- | --- |
| 1 | paeoniflorin | C_23_H_28_O_11_ | 7.06 | 479.1553 | 23.6696 |
| 2 | Galloylalbiflorin or Galloylpaeoniflorin | C_30_H_32_O_15_ | 8.44 | 631.1776 | 14.6134 |
| 3 | Albiflorin | C_23_H_28_O_11_ | 6.53 | 479.1553 | 11.6855 |
| 4 | Galloylalbiflorin or Galloylpaeoniflorin | C_30_H_32_O_15_ | 9.20 | 631.1776 | 10.1254 |
| 5 | benzoylsucrose | C_19_H_25_O_12_ | 11.77 | 445.1346 | 8.24298 |
| 6 | galloylsucrose and isomer | C_19_H_26_O_15_ | 2.40 | 493.1272 | 8.1802 |
| 7 | Hederagenin | C_30_H_48_O_4_ | 21.57 | 471.3474 | 7.98477 |
| 8 | Unknown | C_28_H_28_O_15_ | 22.49 | 603.3543 | 7.88158 |
| 9 | Palbinone isomer | C_22_H_30_O_4_ | 16.54 | 357.2066 | 7.68005 |
| 10 | Unknown | C_21_H_19_O_11_ | 22.88 | 447.2574 | 7.21839 |
| 11 | Oleanolic acid or isomer | C_30_H_48_O_3_ | 25.38 | 455.356 | 6.8385 |
| 12 | Unknown | - | 9.43 | 836.6059 | 6.66364 |
| 13 | Unknown | - | 8.85 | 723.5176 | 6.64994 |
| 14 | Galloylalbiflorin or Galloylpaeoniflorin | C_30_H_32_O_15_ | 8.97 | 631.1776 | 6.61644 |
| 15 | Unknown | C_22_H_18_O_11_ | 19.56 | 457.1858 | 6.3784 |
| 16 | methyl gallate | C_8_H_8_O_5_ | 5.29 | 183.0293 | 6.35201 |
| 17 | Unknown | ­C_26_H_15_O_7_ | 1.41 | 439.0869 | 6.3249 |
| 18 | 1,2,3,4,6-O-pentagalloylglucose | C_41_H_32_O_26_ | 8.39 | 939.111 | 6.28761 |
| 19 | Iso-benzoyloxypaeoniflorin | C_30_H_32_O_12_ | 13.94 | 583.1816 | 6.13231 |
| 20 | Unknown | C_27_H_47_O_14_ | 20.91 | 595.2665 | 6.12123 |

**References**

Hu, Y. F., Pei, Y, M., Wu, H., Xu, Q., Xu, G.B., Jang, L., et al. (2016). Difference analysis of chemical compositions in Moutan Cortex from different origins by UPLC-Q-TOF-MS. *Chin. Tradit. Herbal**. Drugs.* 47, 2984–2991. doi: 10.7501/j.issn.0253-2670.2016.17.005

Li, B., Ge, J., Liu, W., Hu, D., and Li, P. (2021). Unveiling spatial metabolome of Paeonia suffruticosa and *Paeonia lactiflora* roots using MALDI MS imaging. *New*. *Phytol.* 231, 892–902. doi:10.1111/nph.17393

Li, J. H., Zeng, R., Qu, Y., and Huang, L.F. (2017). Rapid identification on chemical constituents in roots of *Paeonia delavayi* var. *lutea* by UPLC-Q-TOF-MS^E^ combined with UNIFI informatics platform. *Chin. Tradit. Herbal. Drugs.* 48,1529–1536. doi: 10.7501/j.issn.0253-2670.2017.08.009

Liu, J., Chen, L., Fan, C. R., Li, H., Huang, M. Q., Xiang, Q., et al. (2015). Qualitative and quantitative analysis of major constituents of Paeoniae Radix Alba and Paeoniae Radix Rubra by HPLC-DAD-Q-TOF-MS/MS *Chin**. J. Chin. Mater. Med.* 40, 1762–1770. doi: 10.4268 /cjcmm20150926

Shi, Y. H., Zhu, S., Ge, Y. W., Toume, K., Wang, Z., Batkhuu, J., et al. (2016). Characterization and quantification of monoterpenoids in different types of peony root and the related *Paeonia* species by liquid chromatography coupled with ion trap and time-of-flight mass spectrometry. *J. Pharm. Biomed. Anal.* 129, 581–592. doi: 10.1016/j.jpba.2016.07.031

Tan, S.Y.，Xiao, X., Piao, S. H., and Li, S. S. (2017). Analysis and identification of water-soluble components of *paeonia lactiflora*. *Chin**. Med. Mat.* 40,94–100. doi: 10. 13863 /j. issn1001-4454. 2017. 01. 023

Wang, Q. L., Liang, Z. T., Peng, Y., Hou, J. L., Wei, S. L., Zhao, Z. Z., et al. (2015). Whole transverse section and specific-tissue analysis of secondary metabolites in seven different grades of root of *Paeonia lactiflora* using laser microdissection and liquid chromatography-quadrupole/time of flight-mass spectrometry. *J*. *Pharm*. *Biomed*. *Anal**.* 103, 7–16. doi: 10.1016/j.jpba.2014.10.020

Zhan, Z. L., Deng, A. P., Kang, L. P., Tang, J. F., Nan, T. G., Chen, T., et al. (2018). Chemical profiling in Moutan Cortex after sulfuring and desulfuring processes reveals further insights into the quality control of TCMs by nontargeted metabolomic analysis. *J*. *Pharm*. *Biomed*. *Anal.*156, 340–348. doi: 10.1016/j.jpba.2018.04.045
