# Supplementary figures and images for: Comparative Elucidation of Age, Diameter, and “Pockmarks” in Roots of Paeonia lactiflora Pall. (Shaoyao) by Qualitative and Quantitative Methods
Source: Front Plant Sci. 2022 Jan 26;12:802196. doi: 10.3389/fpls.2021.802196 (PMC8826210; doi:10.3389/fpls.2021.802196)

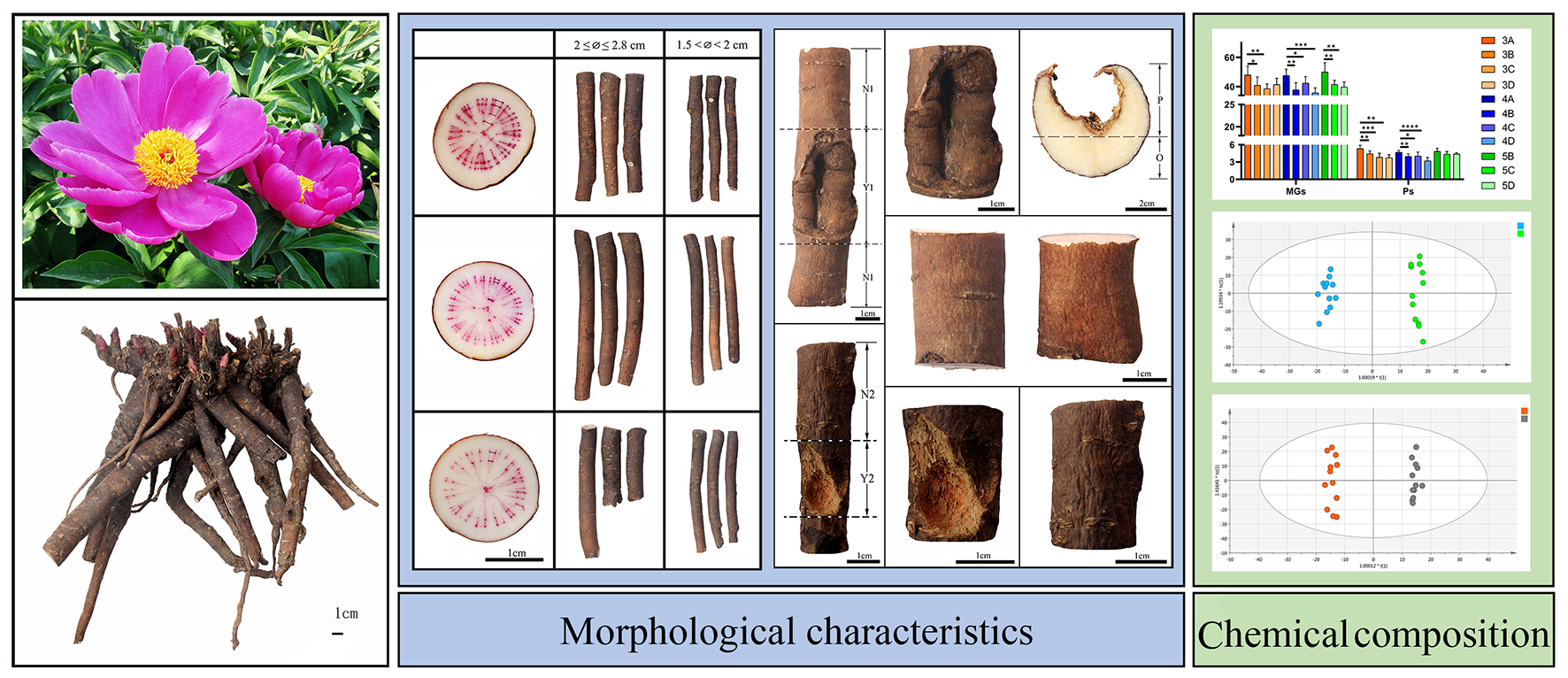

Supplement: Supplementary file 2 [file Image_1.jpg]
